# MRI-derived radiomics assessing tumor-infiltrating macrophages enable prediction of immune-phenotype, immunotherapy response and survival in glioma

**DOI:** 10.1186/s40364-024-00560-6

**Published:** 2024-01-31

**Authors:** Di Chen, Rui Zhang, Xiaoming Huang, Chunxia Ji, Wei Xia, Ying Qi, Xinyu Yang, Lishuang Lin, Jing Wang, Haixia Cheng, Weijun Tang, Jinhua Yu, Dave S. B. Hoon, Jun Zhang, Xin Gao, Yu Yao

**Affiliations:** 1 Department of Neurosurgery, Huashan Hospital, Shanghai Medical College, Fudan University, Shanghai, China; 2 National Center for Neurological Disorders, Shanghai, China; 3 Shanghai Key Laboratory of Brain Function and Restoration and Neural Regeneration, Shanghai, China; 4 Immunology Laboratory, Neurosurgical Institute of Fudan University, Shanghai, China; 5 Shanghai Clinical Medical Center of Neurosurgery, Shanghai, China; 6 Department of Medical Imaging, Suzhou Institute of Biomedical Engineering and Technology, Chinese Academy of Sciences, Suzhou, Jiangsu China; 7 Department of Pathology, Huashan Hospital, Fudan University, Shanghai, China; 8 Department of Radiology, Huashan Hospital, Fudan University, Shanghai, China; 9 Department of Electronic Engineering, Fudan University, Shanghai, China; 10 Department of Translational Molecular Medicine, Saint Johns Cancer Institute, Providence Health Systems, Santa Monica, CA USA; 11 Department of Radiology, Huashan Hospital, State Key Laboratory of Medical Neurobiology, Fudan University, Shanghai, China

**Keywords:** Glioma, Magnetic resonance imaging, Radiomics, Macrophages, Immunotherapy, Prognosis

## Abstract

**Background:**

The tumor immune microenvironment can influence the prognosis and treatment response to immunotherapy. We aimed to develop a non-invasive radiomic signature in high-grade glioma (HGG) to predict the absolute density of tumor-associated macrophages (TAMs), the preponderant immune cells in the microenvironment of HGG. We also aimed to evaluate the association between the signature, and tumor immune phenotype as well as response to immunotherapy.

**Methods:**

In this retrospective setting, total of 379 patients with HGG from three independent cohorts were included to construct a radiomic model named Radiomics Immunological Biomarker (RIB) for predicting the absolute density of M2-like TAM using the mRMR feature ranking method and LASSO classifier. Among them, 145 patients from the TCGA microarray cohort were randomly allocated into a training set (*N*=101) and an internal validation set (*N*=44), while the immune-phenotype cohort (*N*=203) and the immunotherapy-treated cohort (*N*=31, patients from a prospective clinical trial treated with DC vaccine) recruited from Huashan Hospital were used as two external validation sets. The immunotherapy-treated cohort was also used to evaluate the relationship between RIB and immunotherapy response. Radiogenomic analysis was performed to find functional annotations using RNA sequencing data from TAM cells.

**Results:**

An 11-feature radiomic model for M2-like TAM was developed and validated in four datasets of HGG patients (area under the curve = 0.849, 0.719, 0.674, and 0.671) using MRI images of post contrast enhanced T1-weighted (T1CE). Patients with high RIB scores had a strong inflammatory response. Four hub-genes (SLC7A7, RNASE6, HLA-DRB1 and CD300A) expressed by TAM were identified to be closely related to the RIB, providing important evidence for biological interpretation. Only individuals with a high RIB score were shown to have survival benefits from DC vaccine [DC vaccine vs. Placebo: median progression-free survival (mPFS), 10.0 mos vs. 4.5 mos, HR=0.17, *P*=0.0056, 95%CI=0.041-0.68; median overall survival (mOS), 15.0 mos vs. 7.0 mos, HR=0.17, *P* =0.0076, 95%CI=0.04-0.68]. Multivariate analyses also confirmed that treatment by DC vaccine was an independent factor for improved survival in the high RIB score group. However, in the low RIB score group, DC vaccine was not associated with improved survival. Furthermore, a radiomic nomogram based on the RIB score and clinical factors could efficiently predict the 1-, 2-, and 3-year survival rates, as confirmed by ROC curve analysis (AUC for 1-, 2- and 3-year survival: 0.705, 0.729 and 0.684, respectively).

**Conclusions:**

The radiomic model could allow for non-invasive assessment of the absolute density of TAM from MRI images in HGG patients. Of note, our RIB model is the first immunological radiomic model confirmed to have the ability to predict survival benefits from DC vaccine in gliomas, thereby providing a novel tool to inform treatment decisions and monitor patient treatment course by radiomics.

**Supplementary Information:**

The online version contains supplementary material available at 10.1186/s40364-024-00560-6.

## Introduction

Cancer immunotherapy has made significant and promising advancements in extending the survival of patients with various malignancies, achieved through clinical treatments involving immune checkpoint inhibitors (ICIs), DC vaccines and adoptive cell therapies [1]. Unfortunately, not all patients will respond to immunotherapies and only 20–50% of patients with advanced solid tumors derive clinical benefit [2]. Biomarkers are needed to identify patients who are most likely to benefit from this treatment before therapy. The tumor immune microenvironment (TIM) is a complex ecosystem containing various immune factors, which plays a key role in tumor progression and therapeutic response [3]. Several biomarkers associated with immunological status have been discovered with variable success, including tumor mutation burden (TMB) [4], specific genetic mutations [5], expression level of PD-L1 [6] and presence of tumor immune infiltrates [7]. Regarding immune infiltrates, two distinct immune-phenotypes have been described [8]: “hot” tumors infiltrated with abundant immune cells and “cold” tumors characterized by sparse immune infiltration. Therefore, the absolute density of immune infiltration can be a critical indicator of the immune-phenotype. Patients categorized as having “cold” tumors often have a lower overall response to immunotherapy, which indicates that the evaluation of immune cell infiltration is useful in immunotherapy.

Current histological or molecular evaluation of tumor phenotypes requires tissue specimens acquired via surgery. However, a large proportion of patients with advanced tumors are not qualified to receive invasive procedures because of potential morbidity or inoperability. Moreover, when biopsy is feasible, sampling bias induced by intratumor spatial heterogeneity is a considerable problem. Given the dynamic nature of the TIM during tumor progression and treatment delivery, a non-invasive way to assess the tumoral immune infiltrates would be useful to predict the efficacy of immunotherapy without surgical sampling bias, particularly in brain tumors where tumor sampling is not always practical and can cause morbidity.

Radiomics, a quantitative approach originating from radiographic imaging, has achieved remarkable successes in diagnosis, prognosis predicting, and response evaluation in patients with cancer. High-dimensional imaging data referred to radiomics can provide more in-depth characterization than achieved by eye. Through a quantitative radiomics model, rich information of the cellular and molecular properties in the entire tumor region can be visualized. Specific features from images have been confirmed to be linked with tumor phenotype [9], not only containing cancer cell-intrinsic characteristics but also TIM status. Several studies found a strong correlation between imaging features and tumor-infiltrating lymphocytes (TILs) [10] or the expression level of PD-L1 [11]. Moreover, good performance from radiomics in predicting the response to blockade of PD-1/PD-L1 has been observed in lung cancer [12]. Therefore, radiomic signatures are complementary to biopsies and have the advantage of being non-invasive, which allow an unbiased and longitudinal assessment of immune infiltration throughout the treatment course.

Glioma is the deadliest tumor in the central nervous system (CNS) with poor survival time, especially for the high-grade glioma (HGG) [13]. Due to limited selection in treatment, immunotherapy has been used more frequently for glioma patients in clinical trials [14]. Unfortunately, only a subset of patients responds to treatment, which indicates that a predictive biomarker is urgently needed in glioma to improve responses. Studies have demonstrated that subgroups in glioma with more immune infiltrates could benefit from modern immunotherapy approaches [15]. We previously developed a new glioma immune-phenotype using gene expression signatures [16], which identified a “super-cold” group with a lower absolute proportion of tumor-infiltrating immune cells, including M2-like tumor-associated macrophage (TAM), lymphocytes, and neutrophils. MRI-based radiomic features were confirmed to have an association with TIM in gliomas [17–20], especially for TAM infiltration [21]. However, to our knowledge, there is no evidence yet for the potential performance of this kind of immunological radiomics in predicting responses to immunotherapy in gliomas.

In this study, we hypothesize that radiomics will allow for non-invasive evaluation of immune infiltration to determine the immune-phenotypes in HGG, and can lead to the identification of novel predictors for immunotherapy. The primary objective of this study was to develop an MRI-based radiomic signature [Radiomics Immunological Biomarker (RIB)] to predict the classes of M2-like TAM infiltration which significantly changed among immune-phenotypes in our previous study, and to assess the ability of this signature to identify “cold” or “hot” gliomas. The secondary study objective is also to test RIB’s ability to predict clinical response to DC vaccine using HGG patients from a randomized controlled clinical trial.

## Methods and materials

### Study design and participants

**Fig. 1:**
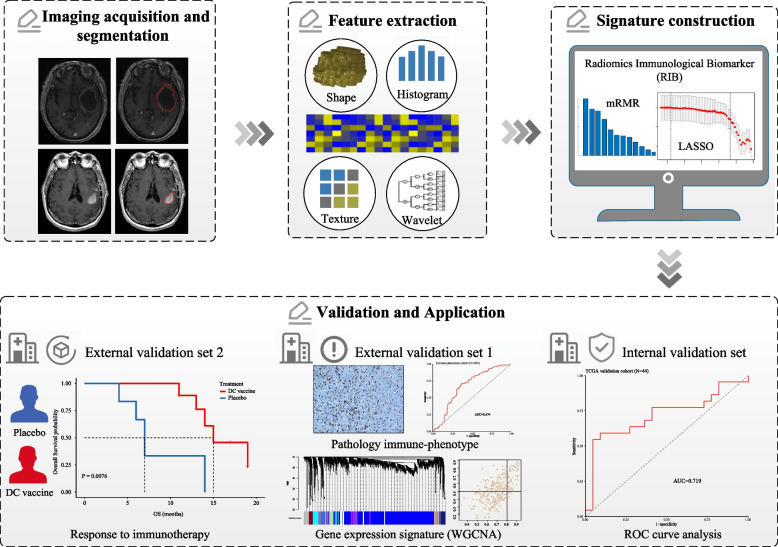
Workflow of radiomics analysis

**Fig. 2:**
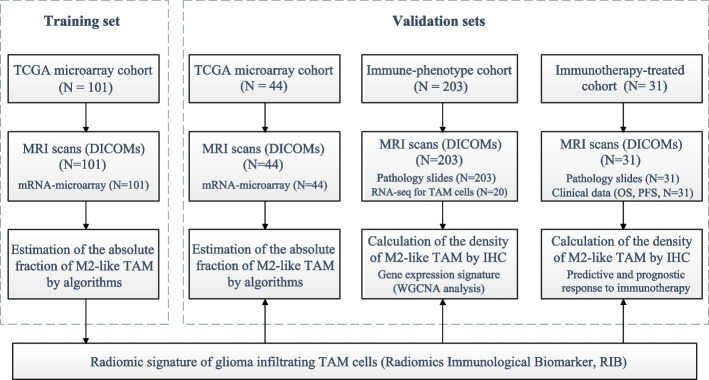
Study design for the development and validation of the radiomic model for the absolute density of M2-like TAM in HGG patients

The overall study design is depicted in Fig. 1. A total of 379 HGG patients from three independent cohorts were enrolled retrospectively to construct and validate a radiomic model (Fig. 2 and Table 1). The TCGA microarray cohort (*n*=145) extracted from The Cancer Imaging Archive (TCIA) was randomly divided into two sets at a ratio of 7:3 using computer-generated random numbers (101 and 44 patients in the training and internal validation sets, respectively) (Additional file 1: Table S1). Two external validation sets included patients from the immune-phenotype cohort (*n*=203) and the immunotherapy-treated cohort (*n*=31) at Huashan Hospital, Fudan University. All of the patients were enrolled following these primary criteria: histologically confirmed high-grade gliomas; the availability of MRI images in DICOM (Digital Imaging and Communications in Medicine) format, specifically T1 contrast gadolinium-enhanced images, performed prior to surgery; and available for gene expression data or immunohistochemistry (IHC) data to obtain absolute level of M2-like TAMs.

**Table 1:** Characteristics of HGG patients in three independent cohorts

**Characteristics**	**TCGA microarray cohort (*N*=145)** Median (range, %)	**Immune-phenotype cohort (*N*=203)** Median (range, %)	**Immunotherapy-treated cohort (*N*=31)** ^a^ Median (range, %)
**Age**	60(14-86)	56(37-77)	51(22-71)
>40	131(90.3%)	175(86.2%)	22(71.0%)
≤40	14(9.7%)	28(13.8%)	9(29.0%)
**Gender**			
Male	92(63.4%)	113(55.7%)	17(54.8%)
Female	53(36.6%)	90(44.3%)	14(45.2%)
**Tumor location**	NA		
Frontal	/	83(40.9%)	16(51.6%)
Non-frontal	/	120(59.1%)	15(48.4%)
**IDH1 mutation**		NA	
Yes	3(2.1%)	19(9.4%)	1(3.2%)
No	116(80.0%)	132(65.0%)	30(96.8%)
NA	26(17.9%)	52(25.6%)	/
**TERT promoter mutation**		NA	
Yes	10(6.9%)	/	14(45.2%)
No	1(0.7%)	/	17(54.8%)
NA	134(92.4%)	/	/

^a^Patients information was extracted from a randomized controlled prospective clinical trial (NCT01567202) whose MRI images are available. Clinicopathological characteristics can be also found in our previous paper^22^

Since MRI images were not available for patients in three other public datasets (CGGA_325 cohort, *N*=139; CGGA_693 cohort, *N*=249; TCGA-RNAseq cohort, *N*=169), a total of 557 HGG patients were included to perform biological validation using gene expression data. Another 20 patients with gene expression data of tumor tissue derived macrophages were extracted from the Brain Disease Immunotherapy Atlas (BDIA) in Huashan Hospital, Fudan University, and the gene expression data was uploaded in The National Omics Data Encyclopedia (NODE, OEP003422).

Patients in the dendritic cell (DC) vaccine immunotherapy-treated cohort were extracted from our published clinical trial (NCT01567202) whose MRI images were accessible [22]. These patients were randomized for treatment with DC vaccine or placebo. In this immunotherapy cohort, progression-free survival (PFS) and overall survival (OS) were defined as the time from the day of random assignment to disease progression and death, respectively. All procedures were approved by the Institutional Review Board of Huashan Hospital, Fudan University (KY2020-009).

### Calculation of the absolute fraction of M2-like TAM

There are two kinds of data resources used to calculate the absolute proportion of M2-like TAM in tumors. For cohorts with gene expression data, including the TCGA microarray cohort, CGGA_325 cohort, CGGA_693 cohort and TCGA-RNAseq cohort, the absolute immune cell fractions for 22 immune cell populations were predicted by CIBERSORT (Cell-type Identification By Estimating Relative Subsets Of RNA Transcripts) and ESTIMATE (Estimation of Stromal and Immune cells in MAlignant Tumour tissues using Expression data) as described previously [16, 23] (Additional file 2: Supplementary methods). IHC staining with CD163 in FFPE tumor tissue sections was used to quantify the density of infiltrating M2-like TAM in two patient cohorts from Huashan Hospital (Additional file 2: Supplementary methods). In the IHC cohort, the patients were defined as high-M2 group (>20%) and low-M2 group (<20%) with a cut-off using the rate of CD133-positive cells (20%) (Additional file 1: Fig. S1). The median value was used to classify the high-M2 group and low-M2 group in the TCGA microarray cohort.

### Image processing and lesion segmentations

All patients underwent MRI examination that included post contrast enhanced T1-weighted (T1CE) prior to tumor resection. The detailed scanning parameters in MRI images of the TCGA dataset can be found on the TCIA website (https://www.cancerimagingarchive.net/). This included MRI images from Huashan Hospital that were generated with scanners manufactured by Siemens (Avanto 1.5T and Verio 3.0T, Germany) or GE (SIGNA EXCITE 3.0T, USA).

Each patient’s images of T1CE were collated and masked by two radiologists (Jing Wang and Jun Zhang) as follows: For each case, the tumor outline was drawn by the first radiologist (JW), and then was checked by the senior radiologist (JZ). The region of the whole enhanced lesion (tumor) was depicted using MITK (Medical Imaging Interaction Toolkit, version 2016.11.0, http://mitk.org/wiki/MITK) in each layer. The nonenhanced area (necrosis) inside of the enhanced lesion was also included. The bleeding lesion inside of the enhanced lesion and the edema area outside of the enhanced lesion were not included in the analysis. All MRI images were bias-field corrected using the N4 bias field correction algorithm from the SimpleITK (Additional file 1: Fig. S2).

Before feature extraction, Z-score normalization built into Pyradiomics (v.2.0.0; http://www.radiomics.io/pyradiomics.html) was performed in T1CE sequences of all the cohorts to reduce the potential effects of scanning parameters, scanners, and vendors.

### Construction of a radiomic model

A simple randomization method was used to divide patients from the TCGA microarray cohort with a ratio of 7:3 into the training set and internal validation set, in which a radiomic model to predict absolute counts of M2-like TAM was developed [Radiomics Immunological Biomarker (RIB)]. Radiomic feature extraction was performed using PyRadiomics. In total, 841 radiomic features were extracted from the T1CE sequence, and were subdivided into eight classes, such as shape features, first order features and wavelet features. The detailed imaging features are described in Supplementary methods. Feature selection was then conducted to eliminate the highly correlated and low reproducible features. After elimination of redundant features, the minimum redundancy maximum relevance (mRMR) method was used to identify the most discriminant feature subset from the remaining features and only the top-ranking features were retained. The least absolute shrinkage and selection operator (LASSO) classifier were used to select the most predictive radiomic features from the top-ranking features and establish a radiomic model for predicting classes of the absolute fraction of M2-like TAM. The classifier was trained using 4-fold cross-validation on the training set to determine the optimal parameter configuration. The receiver operating characteristic curve (ROC) was used to assess the RIB’s ability to distinguish high and low-M2 groups. The optimal cut-off value for RIB was determined using Youden’s index in the training set. More detail on constructing the signature can be found online (Additional file 2: Supplementary methods).

### Association with prognosis and immunotherapy response

We explored the potential association between the radiomic model and immunotherapy efficacy in the immunotherapy-treated cohort. The RIB score was applied to evaluate benefit stratification via comparison of survival time. Within this cohort, patients were stratified into high and low RIB groups based on the median value of the RIB score. Kaplan-Meier curves were employed to assess the disparities in overall survival (OS) and progression-free survival (PFS) between patients who underwent immunotherapy and those who did not within each respective group. In order to evaluate the prognostic value of the RIB score, we built a radiomic nomogram combined with the RIB score and clinicopathological factors to predict the 1-, 2-, and 3-year survival probabilities in the TCGA microarray cohort and immune-phenotype cohort. The ROC curves, calibration curves and decision curves were included to evaluate the potential ability to determine predictive value utility.

### Statistical analysis

The Chi-square test or Fisher’s exact test was used for categorical variables and the t-test or Wilcoxon rank sum test were used for continuous variables between groups, when appropriate. Benjamini and Hochberg was used in GO term, KEGG pathway and GSEA analysis for multiple comparisons. For correlation analysis, the Spearman’s correlation coefficient was selected. Survival curves were generated according to the Kaplan-Meier method and compared by the log-rank test. Univariate and multivariate analyses were completed with the Cox proportional hazards model. All statistical analyses were two-sided performed by SPSS software or R software (version 3.5.1, https://www.r-project.org/). A *P* value <0.05 was considered to be statistically significant.

## Results

### Patient characteristics of the cohorts

The different cohorts and clinicopathological characteristics of enrolled patients (*N*=379) (Figs. 1 and 2) for radiomic analysis are shown in Table 1, whereby 222 (59.0%) were men, with a median age of 57.0 (14.0 to 86.0) years. There were no statistically significant differences in clinicopathologic features between the raining set and the internal validation set (Additional file 1: Table S1). In addition, RNA sequencing data from another 577 HGG patients were extracted from the TCGA, CGGA and BDIA datasets for correlation analysis.

Three independent cohorts were included to establish the radiomic model. The TCGA microarray cohort (*N*=145) was recruited from the TCGA database and were randomly divided into a training set (*N*=101) and an internal validation set (*N*=44) at a ratio of 7:3. The immune-phenotype cohort (*N*=203) and immunotherapy-treated cohort (*N*=31) were recruited from Huashan Hospital, Shanghai as two external validation sets. The patients in the immunotherapy-treated cohort extracted from a randomized controlled clinical trial (NCT01567202) were treated by DC vaccine or placebo. The absolute fraction of M2-like TAM was estimated in the TCGA microarray cohort using the CIBERSORT and ESTIMATE algorithms. For patients in cohorts from Huashan Hospital, the density of M2-like TAM was calculated by IHC staining with CD163 in the tumor slides. Tissue-derived TAM cells were isolated from 20 patients in the immune-phenotype cohort and sequenced to get gene expression, which was used in the radiogenomic analysis (WGCNA).

### Construction and validation of the radiomic model

**Fig. 3:**
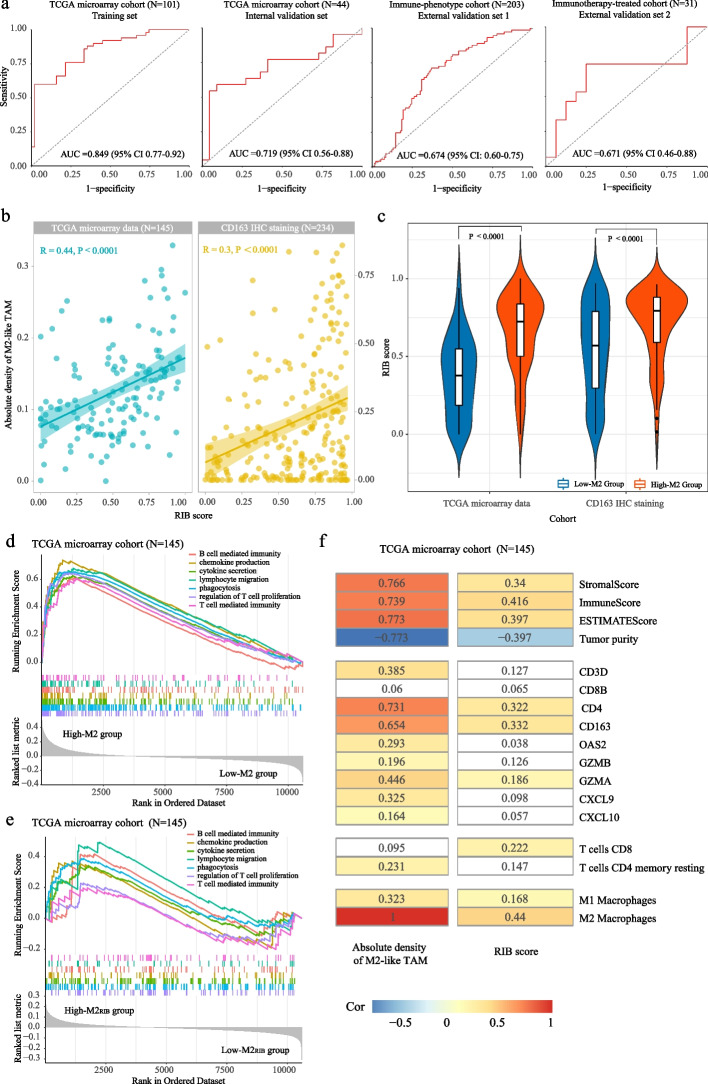
Performance of the RIB model in training and validation sets. **a** ROC curves of the RIB showed the favorable accuracy in four datasets. **b** Significant correlations were found between RIB score and absolute fraction of M2-like TAM. **c** RIB score in high- and low-M2 groups. **d** GSEA enrichment analysis showed more extensive immune responses in patients from high-M2 group. **e** GSEA enrichment analysis also showed more extensive immune responses in patients from high-M2_RIB_ group. **f** Both of the estimated absolute fraction of M2-like TAM (left) and RIB score (right) had positive correlation with immune signatures characterized in hot tumors (eg immunescore) as well as negative correlation with tumor purity characterized in cold tumors. The block diagrams filled with white had no significant *p* values (*p*≥0.05), and the correlation coefficient (R) were embedded in each diagram

In the training set, the 11 most discerning radiomic features from the top-ranking features were selected to formulate the final radiomic signature (RIB). The detailed features and their corresponding coefficients are shown in the supplemental table (Additional file 1: Table S2). The area under the curve (AUC) for RIB was used to classify the high- versus low-M2 group which was 0.849 (95% CI: 0.77 to 0.92) in the training set and 0.719 (95% CI: 0.56 to 0.88) in the internal validation set (Fig. 3a). In the pathologist’s quantification of tumor-infiltrating M2-like TAM in the two external validation sets, the accuracy of RIB was observed with AUCs of 0.674 (95% CI: 0.60 to 0.75) and 0.671 (95% CI: 0.46 to 0.88), respectively (Fig. 3a). In addition, there was a significant (*P* < 0.0001) positive correlation (*R*=0.44, *R*=0.3, respectively) between the RIB score and the absolute fraction of M2-like TAM (Fig. 3b). Figure 3c shows that the RIB score was significantly higher in the high-M2 group than that in the low-M2 group (*P* < 0.0001). All patients were defined into a high-M2_RIB_ group (>0.65) and a low-M2_RIB_ group (<0.65) according to the optimal cut-off (0.65) of the RIB score based on the Youden index in the training set (Additional file 1: Fig. S3). These results suggested that the MRI image-based radiomic model may be useful for non-invasive estimation of tumor-infiltrating immune cells in HGG patients. In order to evaluate the reliability of the model, we randomly split the TCGA microarray cohort two times. The detailed results of each partition were shown in the Table S3 and Fig. S4, which indicated that the model performance remained stable and was not significantly influenced by variations in data partitioning.

### Relationship between the RIB score and immune-phenotype

In our previous study, gliomas with a lower absolute density of M2-like TAM could be defined as “super-cold” tumors which are characterized by limited infiltration of immune cells, including TILs and TAMs. Here, we also found that tumors in the high-M2 group were highly enriched in immune response activity, like phagocytosis, production and secretion of cytokine/chemokine, and T-cell mediated immunity, which are macrophage-specific functions (Fig. 3d). Moreover, analysis showed a significant correlation between the absolute fraction of M2-like TAM and signatures of TIM (Fig. 3f and Additional file 1: Fig. S5). Furthermore, similar results were discovered for RIB scores (Fig. 3e-f), demonstrating that tumors with higher RIB score were analogous to “hot” tumors possessing a stronger intratumoral immune response. In addition, the IDH wild-type (IDHwt) glioma patients from the TCGA microarray cohort were selected to conduct relation analysis, which demonstrated that IDH wild-type glioma patients with higher RIB scores also had more extensive immune response (Additional file 1: Fig. S6).

### Biological annotations of the RIB

**Fig. 4:**
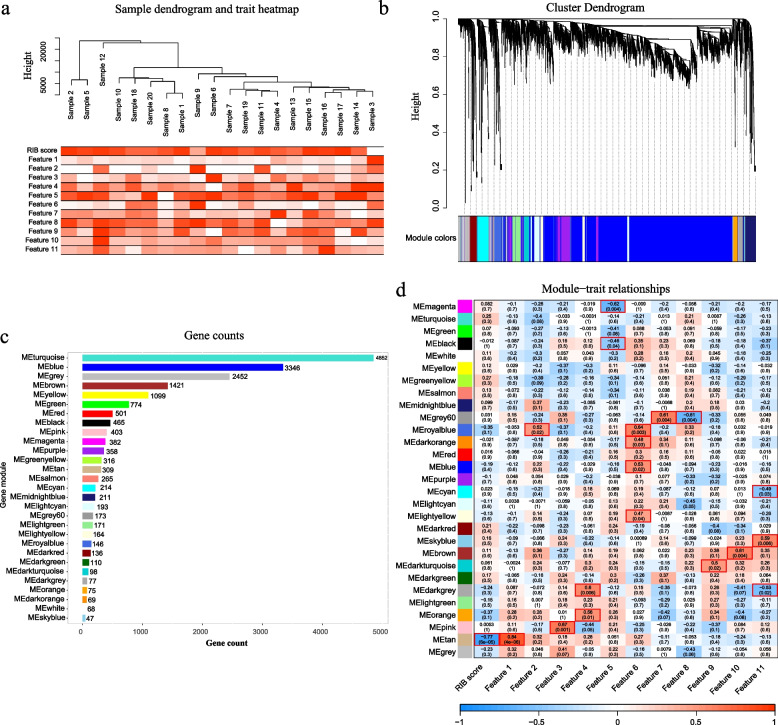
WGCNA identifies key gene modules correlating with RIB and each radiomic feature. **a** No outlier was detected in sample clustering. **b** Gene dendrogram after clustering showed twenty-nine gene modules, and each color indicating one gene module. **c** Counts for each gene module. **d** Module trait relationships were evaluated by correlations between module eigengenes and RIB score or each radiomic feature. *P* < 0.05 represents statistical significance, which were marked by red frame

To explain the intrinsic connection between RIB and immune-phenotype, we generated radiogenomic maps associated RIB with biological processes and pathways by the WGCNA method (Additional file 2: Supplementary methods). Firstly, tumor tissue derived macrophages from 20 HGG patients were sequenced to obtain gene expression data. The genes with low or no differential expression between samples were excluded from WGCNA, whereby 18,895 genes were included for analysis. No outlier samples were observed in the clustering analysis (Fig. 4a). Finally, 29 gene modules revealing different biological functions in TAMs were produced in the coexpression network and shown in the cluster dendrogram (Fig. 4b). Gene counts for each module were noted in the histogram (Fig. 4c). Then, we calculated and mapped the relationship between each module and the RIB score or radiomic features. There were 19 pairwise correlations that were statistically significant (*P*<0.05) in the radiogenomic correlation map (Fig. 4d).

The annotations of these significant gene modules in GO terms (biological processes) and KEGG pathways found three dominating modules named by different colors (pink, brown and tan) correlated with immune processes (Additional file 1: Fig. S7a-b). Of these three modules, the RIB score was strongly correlated with the tan gene module. Hence, the enrichment score for genes in the tan module was calculated using the gene set variation analysis (GSVA) in four cohorts (TCGA microarray cohort, CGGA_325 cohort, CGGA_693 cohort, TCGA-RNAseq cohort) with gene expression data, which showed similar or even better accuracy in predicting the absolute density of M2-like TAM when compared with RIB (Additional file 1: Figs. S7c and S8a). In performing mapping analysis, we found that there was a strong correlation between tan GSVA score and immune-related gene signatures (Additional file 1: Figs. S7d and S8b). Then, we calculated the correlation between the gene and tan module (MM) as well as the gene and RIB (GS) to screen the potential key genes in the tan module. We selected 73 key genes when setting a threshold of GS > 0.5 and MM > 0.8 (Additional file 1: Fig. S9a). In addition, another 447 genes with a significant correlation (cor > 0.2) with the RIB score in the TCGA microarray cohort were selected. Following intersection, 4 hub-genes were identified (*SLC7A7, RNASE6, HLA-DRB1* and *CD300A*) (Additional file 1: Fig. S9b). For further exploration, we elaborated on these hub-genes by correlative analysis (Additional file 1: Fig. S9c) and literature review, which confirmed that they play a key role in regulating immune responses. Radiogenomic correlation and biological validation above revealed at the single-gene level that our RIB model had significant predictive ability to display intratumoral immunological status non-invasively.

### Predictive value of the RIB score for benefits to DC vaccine

**Fig. 5:**
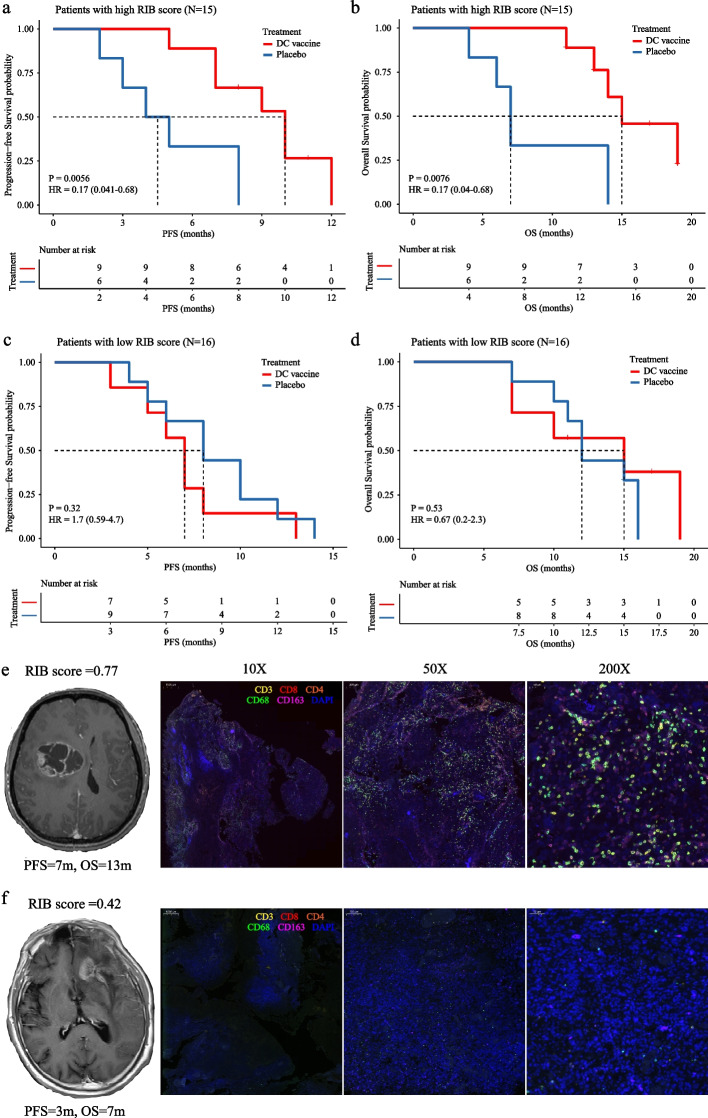
Kaplan-Meier analyses of progression-free survival (PFS) and overall survival (OS) according to RIB score in patients with HGG from immunotherapy-treated cohort. **a** Patients with high RIB score could prolong PFS by treatment from DC vaccine. **b** Patients with high RIB score could prolong OS by treatment from DC vaccine. No significant survival differences of PFS (**c**) or OS (**d**) were found between patients treated by DC vaccine and placebo in subgroup with low RIB score. **e** Multiplexed immunostaining demonstrated one patient with high RIB score was infiltrated by abundant T cells and TAM in TIM, who could be defined as hot immune-phenotype and got an incremental survival from immunotherapy. **f** Few immune cells were observed in another patient with low RIB score, who got attenuated clinical benefit from DC vaccine and was classified into cold immune-phenotype

Previous clinical studies have suggested that immunological signatures in the tumor microenvironment (TME) could predict responses to immunotherapy. We confirmed in this study that HGG patients with high RIB score had abundant immune cell infiltrating and stronger immune response as immunologically “hot” tumors. Therefore, we assessed the predictive value of the RIB score within an immunotherapy-treated cohort. Firstly, the patients were classified into a high RIB score group and a low RIB score group for further analysis based on their median value of RIB score. Within the high RIB group, patients who received DC vaccine immunotherapy had significantly extended survival time compared with patients receiving placebo as expressed in progression-free survival time (10.0 mos vs. 4.5 mos, HR=0.17, *P*=0.0056, 95%CI=0.041-0.68; Fig. 5a) and overall survival time (15.0 mos vs. 7.0 mos, HR=0.17, *P*=0.0076, 95%CI=0.04-0.68; Fig. 5b). The 1-year OS was 77.8% and 33.3% for immunotherapy-treated patients versus placebo-treated patients respectively in the high RIB group. However, for the low RIB group, no significant clinical benefits were obtained for patients receiving DC vaccine in PFS (7.0 mos vs. 8.0 mos, HR=1.7, *P*=0.32, 95%CI=0.59-4.7; Fig. 5c), OS (15.0 mos vs. 12.0 mos, HR=0.67, *P*=0.53, 95%CI=0.2-2.3; Fig. 5d) and 1-year OS (42.9% vs. 44.4%). Multivariate cox regression analysis revealed that immunotherapy remained a strong and independent factor to prolong survival in patients with high RIB score but not in patients with low RIB score (Table 2). In addition, the patients in immunotherapy-treated cohort were divided into two subgroups again using the optimal cut-off value (0.65) obtained from ROC curve analysis to confirm the predictive value of RIB. Both of Kaplan-Meier analysis (Additional file 1: Fig. S10) and multivariate cox regression analysis (Additional file 1: Table S4) also indicated that patients with high RIB score (>0.65) could benefit from DC vaccine, while those with low RIB score (<0.65) were less likely to have survival benefits to DC vaccine.

**Table 2:** Univariate and multivariate cox regression analysis in subgroups from immunotherapy-treated cohort for OS and PFS

**Factor**	**Univariate Cox regression**		**Multivariate Cox regression**	
	**HR (95% CI)**	***P value***	**HR (95% CI)**	***P value***
**OS for patients with high RIB (*N*=15)**				
Age (≤40 vs >40 )	0.87 (0.23-3.4)	0.85	/	/
Gender (Male vs Female)	1 (0.29-3.6)	0.98	/	/
Location (Non-frontal vs Frontal)	1 (0.31-3.3)	0.97	/	/
IDH1^wt^TERT^mt^ (Yes vs No)	1.2 (0.35-3.9)	0.79	0.43 (0.093-2.01)	0.285
Patient (Recurrent vs Newly Diagnosed)	0.82 (0.25-2.7)	0.74	0.96 (0.268-3.41)	0.944
Treatment (DC Vaccine vs Placebo)	0.17 (0.04-0.68)	**0.013**	0.1 (0.018-0.58)	**0.01**
**OS for patients with low RIB (*N*=16)**				
Age (≤40 vs >40 )	0.27 (0.058-1.3)	0.099	0.11 (0.016-0.77)	**0.026**
Gender (Male vs Female)	0.4 (0.12-1.3)	0.13	/	/
Location (Non-frontal vs Frontal)	0.43 (0.13-1.4)	0.16	/	/
IDH1^wt^TERT^mt^ (Yes vs No)	1.7 (0.5-5.6)	0.41	1.33 (0.326-5.42)	0.692
Patient (Recurrent vs Newly Diagnosed)	0.65 (0.21-2.1)	0.47	0.26 (0.053-1.29)	0.099
Treatment (DC Vaccine vs Placebo)	0.67 (0.2-2.3)	0.51	0.84 (0.230-3.05)	0.787
**PFS for patients with high RIB (*N*=15)**				
Age (≤40 vs >40 )	0.52 (0.14-1.9)	0.32	/	/
Gender (Male vs Female)	0.58 (0.18-1.8)	0.36	/	/
Location (Non-frontal vs Frontal)	0.91 (0.29-2.8)	0.86	/	/
IDH1^wt^TERT^mt^ (Yes vs No)	0.78 (0.25-2.4)	0.67	0.36 (0.091-1.40)	0.14
Patient (Recurrent vs Newly Diagnosed)	0.88 (0.28-2.8)	0.83	1.16 (0.349-3.86)	0.808
Treatment (DC Vaccine vs Placebo)	0.17 (0.041-0.68)	**0.013**	0.1 (0.020-0.51)	**0.005**
**PFS for patients with low RIB (*N*=16)**				
Age (≤40 vs >40 )	0.22 (0.047-1)	**0.049**	0.19 (0.033-1.1)	0.07
Gender (Male vs Female)	0.93 (0.33-2.6)	0.89	/	/
Location (Non-frontal vs Frontal)	0.4 (0.13-1.2)	0.1	0.3 (0.047-1.9)	0.205
IDH1^wt^TERT^mt^ (Yes vs No)	1.4 (0.48-4.3)	0.52	1.25 (0.315-5.0)	0.749
Patient (Recurrent vs Newly Diagnosed)	1.5 (0.51-4.3)	0.47	0.6 (0.102-3.5)	0.572
Treatment (DC Vaccine vs Placebo)	1.7 (0.59-4.7)	0.34	2.83 (0.839-9.6)	0.094

To further elucidate the credibility of the RIB score to predict clinical response to DC vaccine, we showed two representative cases receiving DC vaccine treatment whose tumor tissues were accessible for assessment in Fig. 5. For the first patient in the high RIB score group (Fig. 5e), abundant infiltrating of TAMs and TILs were detected in the TME pre-immunotherapy assessment, demonstrating that extratumoral immune cells could migrate into tumor more easily which underlies the function of the DC vaccine. In contrast, few immune cells were observed in tumor of another patient from the low RIB group (Fig. 5f), indicating that the TME was weak in the ability to recruit immune cells, which resulted in the poor efficacy of the DC vaccine treatment. Notably, the first patient with a high RIB score (RIB=0.77) had a longer survival time (PFS, 7.0 mos vs. 3.0 mos; OS, 13.0 mos vs. 7.0 mos) than the second patient with a low RIB score (RIB=0.42). Taken together, these data suggest that the RIB signature is a strong predictive biomarker to noninvasively identify patients who benefit from DC vaccine.

### Prognostic value of RIB in the nomogram model for estimating outcome

**Fig. 6:**
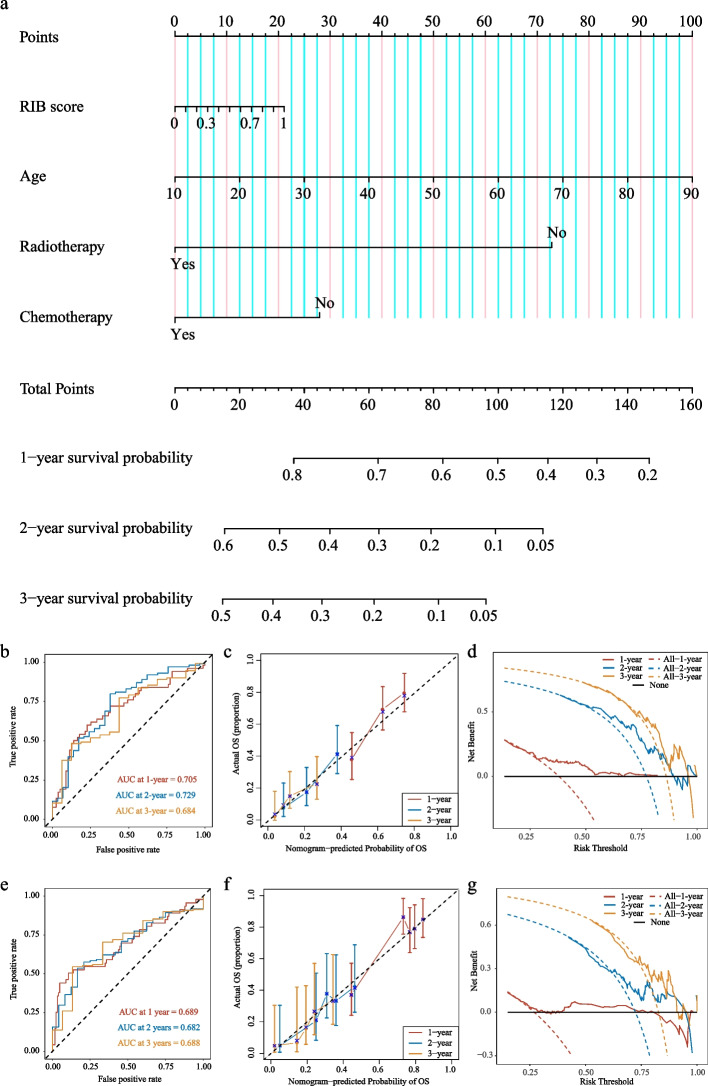
Nomogram, ROC curves, calibration curves, and decision curves to estimate survival probability at 1, 2, and 3-year in the radiomic nomogram. **a** Development of the radiomic nomogram for estimating survival probability integrated with RIB score and clinicopathological information in the primary cohort (TCGA microarray cohort, *N*=145). The ROC curves for the radiomic nomogram in the primary (**b**) and validation (**e**, immune phenotype cohort, *N*=203) cohorts. The calibration curves in the primary (**c**) and validation (**f**) cohorts, the error bars were defined as s.e.m., which represent the 95% CI. The decision curves in the primary (**d**) and validation (**g**) cohorts

Immunological signatures were previously confirmed to be associated with the survival of glioma patients. Therefore, a radiomic nomogram integrated with the RIB score (Fig. 6) was constructed using the TCGA microarray cohort (*N*=145) followed by being validated in the immune-phenotype cohort (*N*=203). ROC curves in the radiomic nomogram showed good efficacy in predicting survival probability at 1, 2, and 3-years in the primary cohort (Fig. 6b and e). Good agreement between the estimations and the actual outcomes of the radiomic nomogram in the primary and validation cohorts were shown in the calibration curves (Fig. 6c and f). The decision curves (Fig. 6d and g) had advantages across almost the entire range of reasonable threshold probabilities which verified good performance for the radiomic nomogram. The radiomic nomogram also had good performance for C-index values in the primary and validation cohorts (Additional file 1: Table S5). These data indicated that the radiomic nomogram based on the radiomic signature and clinicopathologic factors were useful for clinical outcome prediction in HGG patients.

## Discussion

Although TIM has been determined to be an important factor in immunotherapy, noninvasive predictors based on radiographs have only recently been developed as biomarkers for immunotherapy in solid tumors [10]. To our knowledge, no radiomic biomarker has been verified for immunotherapy in gliomas. Considering that T1CE MRI images are used commonly for diagnosis of HGG, we developed and validated a radiomic signature named RIB to predict classes of M2-like TAM by radiomic analysis based on the T1CE sequence. Moreover, we also assessed the association between the radiomic model, immunological signatures, and immunotherapy efficacy. Our RIB showed credible predictive ability and reproducibility across different cohorts with favorable performance (AUC=0.849, 0.719, 0.674, 0.671 in four sets respectively) to predict classes of absolute fraction of TAM. Additionally, this radiomic model was in close relationship with signatures of the immune microenvironment, which showed that patients with higher RIB score acted as “hot” tumors whereby the TME had more immune cells and stronger immune response. Furthermore, we were able to confirm the prognostic value of the RIB signature because a radiomic nomogram combining the radiomic signature with clinicopathological characteristics had a good ability to predict survival time. Of note, we demonstrated for the first time that the radiomic model could predict clinical responses to immunotherapy in glioma patients treated by DC vaccine.

The histopathological analysis of resected tumors is indispensable in the current evaluation of the TIM, which can only be carried out in patients undergoing surgery or biopsy. Moreover, the histological evaluation can be complicated by spatial heterogeneity and temporal evolution of the TIM as studies have recently shown [24]. A radiomic model based on radiographical imaging has been demonstrated to be a desirable biomarker in solid neoplasms to reflect tumor features non-invasively prior to surgery and throughout the treatment course, such as genomic mutations, metastasis, and therapeutic responses. For the TIM, previous studies have established radiomic models to predict immune signatures in solid tumors [25, 26], including the expression level of immune checkpoint molecules such as PD-L1 [11] and counts of tumor-infiltrating immune cells such as CD8^+^ T cells [10]. However, a radiomic model predicting signature of TIM in gliomas is still in the early stage of development. Xuanwei Zhang et al established several radiomic models [18–20] to extrapolate immune signatures in gliomas, such as the estimated infiltration of immune cells. There is more importance in the assessment of glioma using radiomics because elective surgery or biopsy is not often practical, which indicates that information from images becomes a more critical clinical tool for treatment decisions in brain tumors. In a study, Chul‑Kee Park et al [17] verified that radiomic signatures could predict the real fraction of immune cells in gliomas. However, these previous studies had limitations, including a small number of patients or a lack of clinical data to evaluate performance for predicting response to immunotherapy. These proof-of-principle studies were conducted without biological interpretation utilizing transcriptomic analysis of immune cells. Here, we validated our RIB model for absolute counts of M2-like TAM in a large dataset of 379 patients from three independent cohorts, and also provided functional annotation using RNA sequencing data from 577 patients from four independent cohorts. In addition, we confirmed that our radiomic model will be useful to facilitate longitudinal assessment of clinical benefits throughout immunotherapy-treated course in a previous clinical trial for HGG patients using DC vaccine [22].

To our knowledge, our RIB is the first kind of immunological radiomic model verified to be a non-invasive predictor of response to immunotherapy in gliomas. In patients with glioma, biomarkers for selecting candidates suitable for immunotherapy remain ambiguous. In our previous study, we found that glioma patients with high expression of B7-H4 had no survival benefits from DC vaccine, one of the main forms of immunotherapies, whereas others having low expression benefited with improved survival time from immunotherapy [22]. Meanwhile, Genova C et al [27] also confirmed that expression level of B7-H4 was a risk factor in lung cancer patients treated with ICIs. Furthermore, the patients in the glioma subgroup defined by high expression levels of B7-H4 could be identified as “super-cold” tumors in our data [16]. “Super-cold” tumor patients were detected with lower absolute counts of immune cells in the TME, including M2-like TAM, indicating that these patients may be insensitive to existing forms of immunotherapy. The absolute fraction of M2-like TAM had a significant positive correlation with immune infiltrations and was also reduced markedly in “super-cold” tumors, though it is the dominant immunosuppressive cell type in the glioma microenvironment [28]. Although previous studies have constructed immunological radiomics in gliomas but no patients treated with immunotherapy were included. Several studies found significant association between macroscopical imaging features and survival in gliomas patients treated by immunotherapy [29–31]. Recently, a radiomic model has been developed and trained by E. George to predict survival time in glioma cohort treated by anti-PD-L1 antibody [32]. However, this radiomics model was only verified to have a favorable outcome prediction, and there was no ability to detect differences in radiomic scores between responders and non-responders; therefore, it could not identify patients who could benefit from immunotherapy. In our study, we showed that the RIB signature could identify glioma patients who are more likely to benefit in immunotherapy from our randomized controlled prospective clinical trial. Specifically, patients with a high RIB score were able to derive survival benefit from DC vaccine compared to patients treated by placebo. Patient group having a high RIB score had more intense immune response in the TME while RIB score also had meaningful positive correlation with immune infiltration (immunescore predicted by ESTIMATE). This also predicted that it would be unlikely that low-RIB HGG would respond to DC vaccine. Therefore, the RIB model is a composite measure of TIM that likely not only depict absolute fraction of M2-like TAM but also represents specific network of local immunity in the tumor. The density of M2-like TAM has been associated with a poor prognosis of glioma patients in previous studies [33], and we provided further evidence from our cohorts that combing the radiomic signature with clinicopathological information in a radiomic nomogram had great efficacy to predict survival probability in HGG patients without immunotherapy. This may be because that high-dimensional data from normalized images could provide additional characterizations, which also allowed radiomics to be less confused by patient distributions from different cohorts.

In order to explain why our RIB signature could decode intrinsic characteristics of the gliomas TIM and predict immunotherapy responses, we uncovered gene clusters in close relationship with the RIB score by WGCNA analysis using RNA sequencing data of patient-derived TAM cells. Genes enriched in pathways related to macrophage-specific immune function, including antigen processing and presentation, T cell response and phagocytosis, were highly correlated to RIB. Then, we extracted the top four hub-genes positively related to RIB score to expound biological annotation, including *SLC7A7, RNASE6, HLA-DRB1* and *CD300A*, all of which have been confirmed to play a key role in TIM especially in biology triggered by TAM [34–37]. CD300A belongs to the CD300 receptor family, constituting a type I transmembrane protein which was involved in pathways for immune suppression and activation, such as its role in regulating the chemotaxis of immune cells. HLA antigens are indispensable in the antigen presentation process, participating in the activation of T lymphocytes. HLA-DRB1 is one of the gene phenotypes of HLA-DR, classified as an HLA class II antigen, which is primarily associated with the activation of CD4+ T-cells. Studies demonstrated that the expression level of the HLA-DRB1 were correlated with characteristics of immune microenvironment. RNASE6 belongs to the ribonuclease family and has been confirmed to be involved in the polarization of M2-likeTAM. The gene mutations of the amino acid transport protein SLC7A7 have been confirmed to be the risk factors for the development of tumors, including glioblastoma. Additionally, these mutations in SLC7A7 gene were associated with immune functions, such as the regulation of inflammatory responses by macrophages. Generally, this was the first study in gliomas to provide functional annotation for radiomics in a particular population of immune cells.

This study has some limitations. First, the RIB was constructed retrospectively, which renders it susceptible to possible selection bias. However, the negative effect was attenuated when we have included large independent cohorts of patients from multicenters to investigate our findings and validate the reproducibility. Moreover, patients in a randomized controlled, double blind, prospective clinical trial published previously were included in the analysis to ensure the ability of RIB to predict clinical benefits from immunotherapy. Second, the model was trained and validated using absolute cell counts of M2-like TAM from different data sources. The estimated cell counts predicted by hundreds of genes were used in the training set and internal validation set, while the cell fraction was calculated in FFPE sections stained with CD163 by IHC in two external validation sets. Furthermore, the cut-off values to define the low- or the high-M2 group in the four sets were also different. The contradictions mentioned above may contribute to the lower performance in external validation sets (AUC=0.674 and 0.671). However, we found a significant positive correlation between the RIB score and the density of CD163-positive cells, as well as a higher RIB score in the high-M2 group defined by CD163, which showed a favorable agreement between the radiomic score and pathology. Another prospective cohort with fresh tissues to calculate real cell counts is needed to validate this study in the future.

## Conclusion

In this work, we identified an MRI-based radiomic model named RIB that allows the non-invasive evaluation of the HGG immune microenvironment, specifically the absolute fraction of M2-like TAM. Moreover, we confirmed for the first time in glioma patients that the immunological radiomics could be used to predict and monitor the response to DC vaccine, one of the immunotherapies. Furthermore, we assessed the biological fundament of the non-invasive biomarker and verified it to be correlated with the immune response triggered by macrophages. Although large-scale prospective studies are needed for validation, our study has provided extensive evidence that MRI-based radiomic biomarkers may serve as non-invasive predictors of clinical benefits from immunotherapy in glioma patients.

### Supplementary Information

**Additional file 1:** **Fig. S1.** Representative examples of class of absolute density of M2-like TAM calculated by IHC staining with CD163. **Fig. S2.** MRI images were normalized using the N4 bias field correction algorithm. **Fig. S3.** ROC curves for random partitions in the training set and internal validation set from TCGA microarray cohort showed the model is reliable. **Fig. S4.** Distribution of RIB score in TCGA microarray cohort and IHC cohort. **Fig. S5.** Estimated absolute fraction of M2-like TAM had positive correlation with immune signatures characterized in hot tumors (eg immunescore) as well as negative correlation with tumor purity characterized in cold tumors. **Fig. S6.** Biological analyses in IDH-wildtype glioma patients from TCGA microarray cohort showed positive relationship between RIB score and ''hot'' immunological microenvironment. **Fig. S7.** Gene module (tan) in close relationship with the RIB score was enriched in immune response process especially triggered by macrophages. **Fig. S8.** Gene module (tan) was in close relationship with hot immune-phenotype. **Fig. S9.** Gene module (tan) was closely related with hot immune-phenotype. **Fig. S10.** Kaplan-Meier analyses of progression-free survival (PFS) and overall survival (OS) according to the optimal cut-off value (0.65) in patients from immunotherapy-treated cohort. **Table S1.** Characteristics of patients in training set and internal validation set from TCGA microarray cohort. **Table S2.** The coefficient of each imaging feature in the signature. **Table S3.** Evaluation metrics of the results from random partitions in TCGA microarray cohort. **Table S4.** Univariate and multivariate cox regression analysis in subgroups predicted by the optimal cut-off value (0.65) of RIB in immunotherapy-treated cohort for OS and PFS. **Table S5.** The performances of the radiomic nomogram in the different cohorts.

**Additional file 2.** Supplementary Methods.

## Data Availability

The datasets used and analyzed in the current study are available from the corresponding author on reasonable request. The radiomics model will also be available on reasonable request.

## Abbreviations

TAM: Tumor-associated macrophage
AUC: Area under the curve
PFS: Progression-free survival
OS: Overall survival
ICI: Immune checkpoint inhibitors
TIM: Tumor immune microenvironment
TMB: Tumor mutation burden
TIL: Tumor-infiltrating lymphocyte
HGG: High-grade glioma
CNS: Central nervous system
TCIA: The Cancer Imaging Archive
T1CE: T1 contrast gadolinium-enhanced
DC: Dendritic cell
CIBERSORT: Cell-type Identification By Estimating Relative Subsets Of RNA Transcripts
ESTIMATE: Estimation of Stromal and Immune cells in MAlignant Tumour tissues using Expression data
IHC: Immunohistochemistry
mRMR: Minimum redundancy maximum relevance
LASSO: Least absolute shrinkage and selection operator
ROC: Receiver operating characteristic curve
TCGA: The Cancer Genome Atlas
CGGA: Chinese Glioma Genome Atlas
WGCNA: Weighted gene co-expression network analysis
GSVA: Gene set variation analysis
DICOMS: Digital Imaging and Communications in Medicine
