# Supplementary material for: MRI-derived radiomics assessing tumor-infiltrating macrophages enable prediction of immune-phenotype, immunotherapy response and survival in glioma
Source: Biomark Res. 2024 Jan 31;12:14. doi: 10.1186/s40364-024-00560-6 (PMC10829320; doi:10.1186/s40364-024-00560-6)
Supplement: Supplementary file 1 — Additional file 1: Fig. S1. Representative examples of class of absolute density of M2-like TAM calculated by IHC staining with CD163. Fig. S2. MRI images were normalized using the N4 bias field correction algorithm. Fig. S3. ROC curves for random partitions in the training set and internal validation set from TCGA microarray cohort showed the model is reliable. Fig. S4. Distribution of RIB score in TCGA microarray cohort and IHC cohort. Fig. S5. Estimated absolute fraction of M2-like TAM had positive correlation with immune signatures characterized in hot tumors (eg immunescore) as well as negative correlation with tumor purity characterized in cold tumors. Fig. S6. Biological analyses in IDH-wildtype glioma patients from TCGA microarray cohort showed positive relationship between RIB score and ''hot'' immunological microenvironment. Fig. S7. Gene module (tan) in close relationship with the RIB score was enriched in immune response process especially triggered by macrophages. Fig. S8. Gene module (tan) was in close relationship with hot immune-phenotype. Fig. S9. Gene module (tan) was closely related with hot immune-phenotype. Fig. S10. Kaplan-Meier analyses of progression-free survival (PFS) and overall survival (OS) according to the optimal cut-off value (0.65) in patients from immunotherapy-treated cohort. Table S1. Characteristics of patients in training set and internal validation set from TCGA microarray cohort. Table S2. The coefficient of each imaging feature in the signature. Table S3. Evaluation metrics of the results from random partitions in TCGA microarray cohort. Table S4. Univariate and multivariate cox regression analysis in subgroups predicted by the optimal cut-off value (0.65) of RIB in immunotherapy-treated cohort for OS and PFS. Table S5. The performances of the radiomic nomogram in the different cohorts. [file 40364_2024_560_MOESM1_ESM.docx]

**Table S1:** **Characteristics of patients in training set and internal validation set from TCGA microarray cohort**

| **Characteristics** | **TCGA microarray cohort training set (n=101)** Median (range, %) | **TCGA microarray cohort internal validation set (n=44)**  Median (range, %) | **P value** |  |
| --- | --- | --- | --- | --- |
|  |  |  |  |  |
| **Age** | 61（14-86） | 57（17-78） | 0.125 |  |
| ＞40 | 94（90.1%） | 37（84.1%） |  |  |
| ≤40 | 7（9.9%） | 7（15.9%） |  |  |
| **Gender** |  |  | 0.574 |  |
| Male | 66（63.3%） | 26（59.1%） |  |  |
| Female | 35（36.7%） | 18（40.9%） |  |  |
| **IDH1 mutation** |  |  | 0.992 |  |
| Yes | 2（2.0%） | 1（2.3%） |  |  |
| No | 81（80.2%） | 35（79.5%） |  |  |
| NA | 18（17.8%） | 8（18.2%） |  |  |
| **TERT promoter mutation** |  |  | 1 |  |
| Yes | 5（5.0%） | 5（11.4%） |  |  |
| No | 1（0.9%） | 0（0%） |  |  |
| NA | 95（94.1%） | 39（88.6%） |  |  |

**Table S2: The coefficient of each imaging feature in the signature**

| **Feature** | **Name** | **coefficient** |
| --- | --- | --- |
| (Intercept) | (Intercept) | -50.76137481 |
| Feature 1 | original_firstorder_Kurtosis | -0.990003266 |
| Feature 2 | original_glszm_SmallAreaLowGrayLevelEmphasis | -1.479841837 |
| Feature 3 | wavelet.LHL_glcm_MaximumProbability | -7.026753404 |
| Feature 4 | wavelet.LHL_glcm_SumEntropy | 34.66242731 |
| Feature 5 | wavelet.LHH_glszm_GrayLevelVariance | 3.363962077 |
| Feature 6 | wavelet.HLL_firstorder_Kurtosis | -0.192494379 |
| Feature 7 | wavelet.HLL_glszm_ZoneEntropy | 0.695654996 |
| Feature 8 | wavelet.HLH_glszm_GrayLevelVariance | 8.607859503 |
| Feature 9 | wavelet.HLH_glszm_SmallAreaEmphasis | -1.87754845 |
| Feature 10 | wavelet.HHL_firstorder_Median | 256.45401 |
| Feature 11 | wavelet.HHH_firstorder_Skewness | 2.429873174 |

**Table S3: Evaluation metrics of the results from random partitions in TCGA microarray cohort**

|  | **AUC of the training set** | **AUC of the** **internal validation set** | |
| --- | --- | --- | --- |
| RIB model | 0.849 | | 0.719 |
| Random partition 1 | 0.821 | | 0.726 |
| Random partition 2 | 0.839 | | 0.721 |
| Mean±standard deviation | 0.836±0.011 | | 0.722±0.003 |

**Table S4: Univariate and multivariate cox regression analysis in subgroups predicted by the optimal cut-off value (0.65) of RIB in immunotherapy-treated cohort for OS and PFS**

| **Factor** | **Univariate Cox regression** | | **Multivariate Cox regression** | |
| --- | --- | --- | --- | --- |
|  | **HR (95% CI)** | **P *value*** | **HR (95% CI)** | **P *value*** |
| **OS for patients with high RIB score (＞0.65) (N=19)** | | | | |
| Age (≤40 vs ＞40 ) | 0.7 (0.19-2.5) | 0.59 | / | / |
| Gender (Male vs Female) | 0.82 (0.29-2.4) | 0.72 | / | / |
| Location (Non-frontal vs Frontal) | 1 (0.31-3.3) | 0.8 | / | / |
| IDH1^wt^TERT^mt^ (Yes vs No) | 0.87 (0.32-2.4) | 0.79 | 0.93 (0.292-2.93) | 0.896 |
| Patient (Recurrent vs Newly Diagnosed) | 0.73 (0.26-2) | 0.55 | 0.59 (0.191-1.82) | 0.358 |
| Treatment (DC Vaccine vs Placebo) | 0.31 (0.11-0.92) | **0.034** | 0.28 (0.088-0.88) | **0.029** |
| **OS for patients with low RIB score (＜0.65) (N=12)** | | | | |
| Age (≤40 vs ＞40 ) | 0.35 (0.068-1.8) | 0.2 | / | / |
| Gender (Male vs Female) | 0.33 (0.075-1.5) | 0.14 | / | / |
| Location (Non-frontal vs Frontal) | 0.35 (0.079-1.6) | 0.17 | / | / |
| IDH1^wt^TERT^mt^ (Yes vs No) | 1.1 (0.22-5.4) | 0.93 | 1.18 (0.176-7.9) | 0.865 |
| Patient (Recurrent vs Newly Diagnosed) | 0.66 (0.16-2.8) | 0.57 | 0.68 (0.135-3.4) | 0.634 |
| Treatment (DC Vaccine vs Placebo) | 0.38 (0.074-1.9) | 0.24 | 0.35 (0.063-2.0) | 0.235 |
| **PFS for patients with high RIB score (＞0.65) (N=19)** | | | | |
| Age (≤40 vs ＞40 ) | 0.46 (0.13-1.6) | 0.23 | / | / |
| Gender (Male vs Female) | 0.55 (0.2-1.5) | 0.23 | / | / |
| Location (Non-frontal vs Frontal) | 0.85 (0.32-2.3) | 0.75 | / | / |
| IDH1^wt^TERT^mt^ (Yes vs No) | 0.88 (0.33-2.4) | 0.8 | 0.63 (0.23-1.77) | 0.383 |
| Patient (Recurrent vs Newly Diagnosed) | 0.9 (0.33-2.4) | 0.84 | 0.65 (0.23-1.85) | 0.425 |
| Treatment (DC Vaccine vs Placebo) | 0.26 (0.084-0.83) | **0.023** | 0.21 (0.06-0.71) | **0.012** |
| **PFS for patients with low RIB score (＜0.65) (N=12)** | | | | |
| Age (≤40 vs ＞40 ) | 0.23 (0.047-1.11) | 0.065 | 0.14 (0.016-1.2) | 0.07 |
| Gender (Male vs Female) | 1.1 (0.34-3.8) | 0.83 | / | / |
| Location (Non-frontal vs Frontal) | 0.34 (0.086-1.3) | 0.12 | / | / |
| IDH1^wt^TERT^mt^ (Yes vs No) | 1.3 (0.32-5) | 0.74 | 2.57 (0.309-21.3) | 0.383 |
| Patient (Recurrent vs Newly Diagnosed) | 1.9 (0.53-6.7) | 0.33 | 1.26 (0.199-8.0) | 0.804 |
| Treatment (DC Vaccine vs Placebo) | 1.4 (0.42-4.7) | 0.57 | 1.79 (0.403-8.0) | 0.444 |

**Table S5: The performances of the radiomic nomogram in the different cohorts**

| **Cohort** | **C-index (95% CI)** |  |
| --- | --- | --- |
|  |  |  |
| Primary cohort (TCGA microarray cohort, n=145) | 0.671 (0.614-0.729) |  |
| Validation cohort (Immune phenotype cohort, n=100) | 0.669 (0.610-0.728) |  |


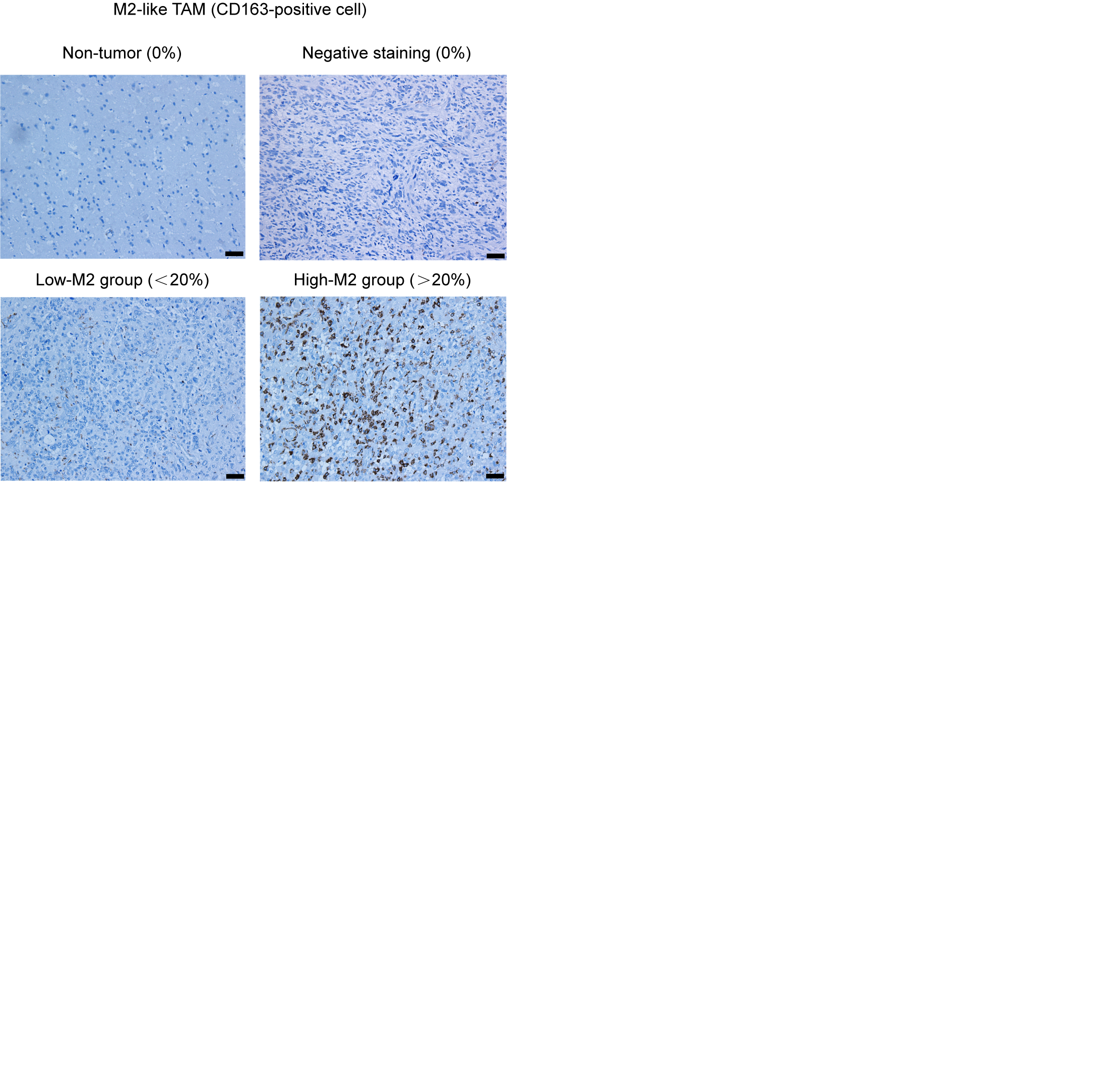


**Fig. S1** Representative examples of class of absolute density of M2-like TAM calculated by IHC staining with CD163.


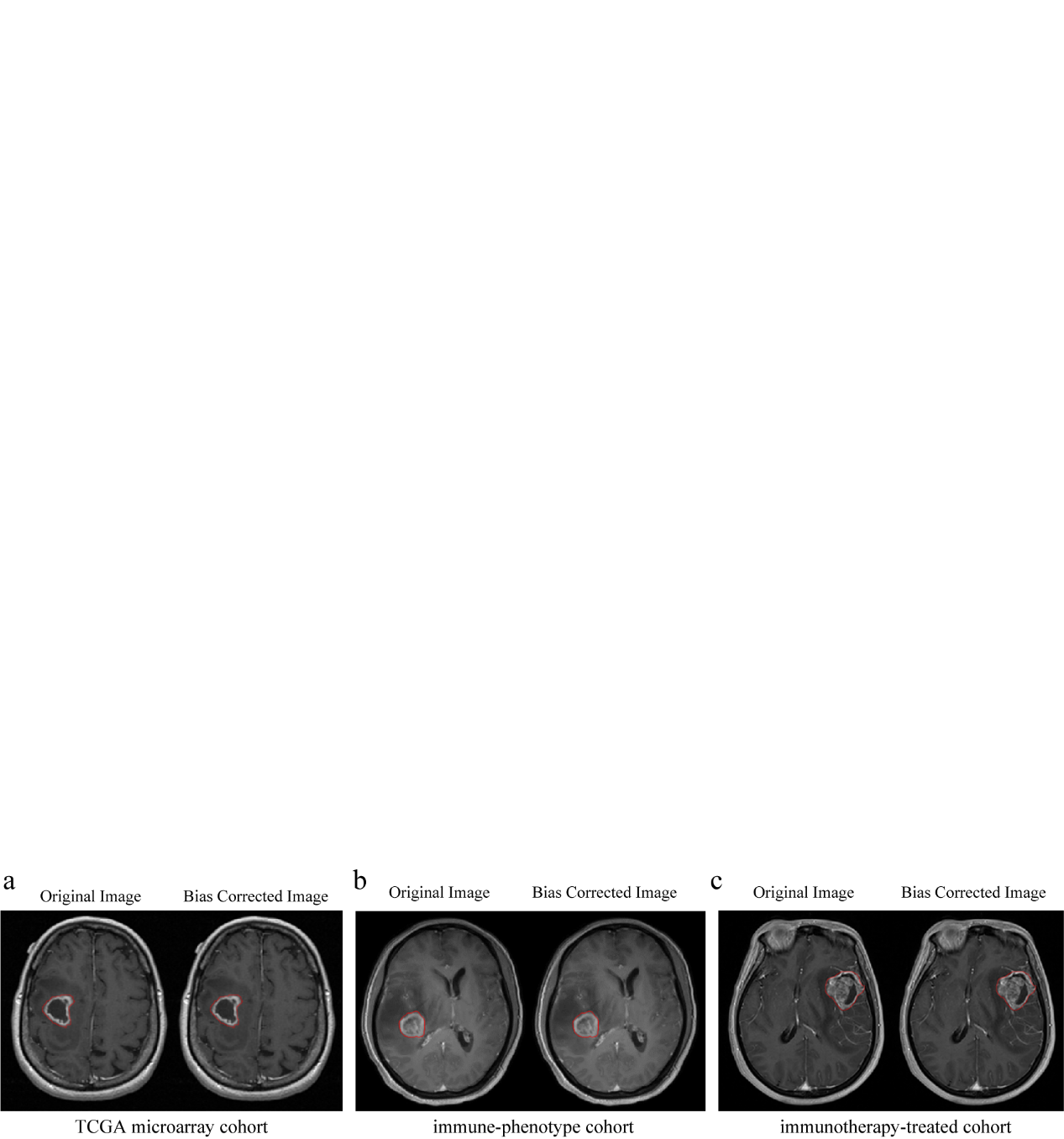


**Fig. S2** MRI images were normalized using the N4 bias field correction algorithm.

(a) One patient from the TCGA microarray cohort; (b) One patient from the immune-phenotype cohort; (c) One patient from the immunotherapy-treated cohort with DC vaccine.


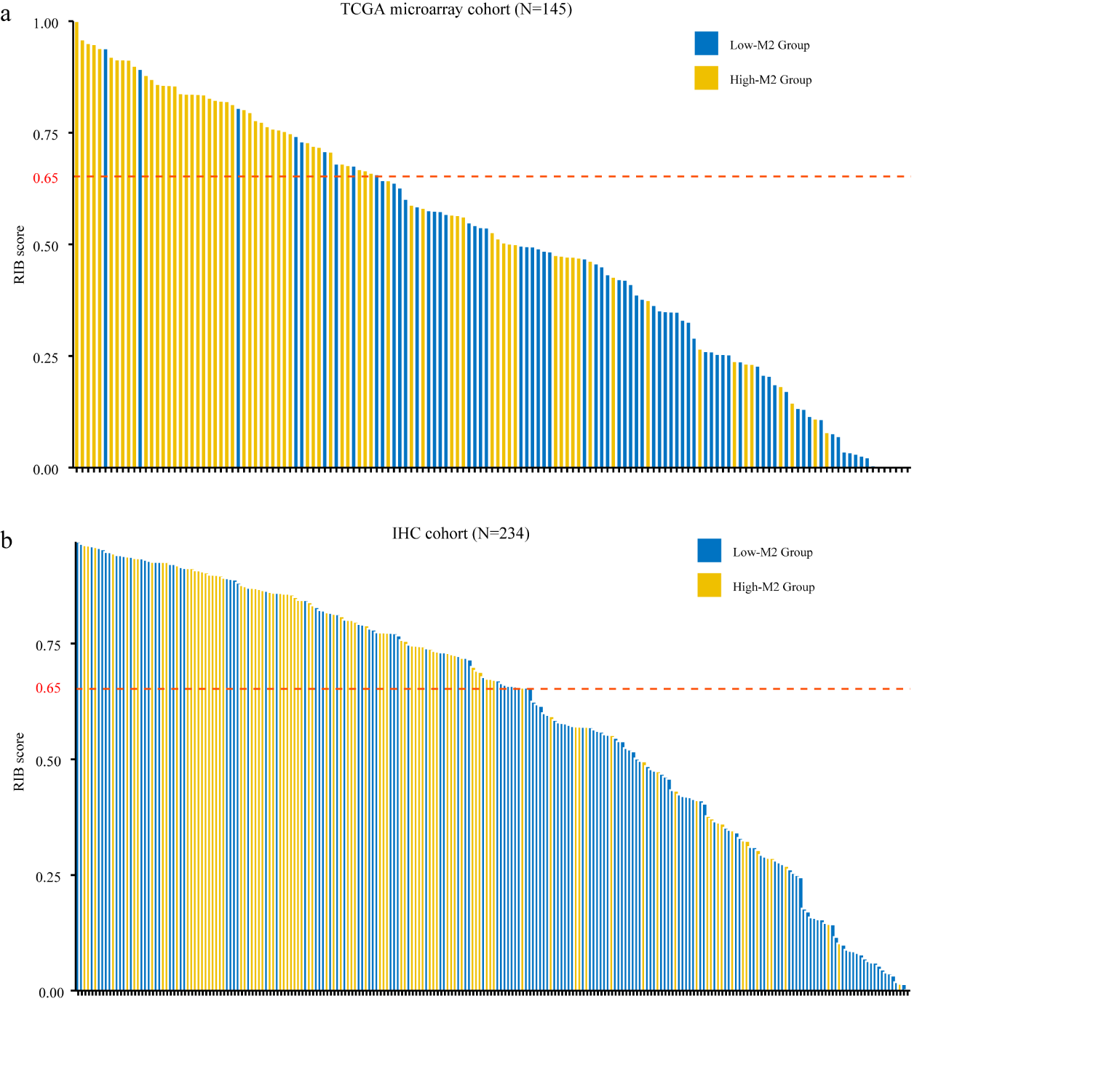


**Fig. S3** Distribution of RIB score in TCGA microarray cohort (a) and IHC cohort (b).


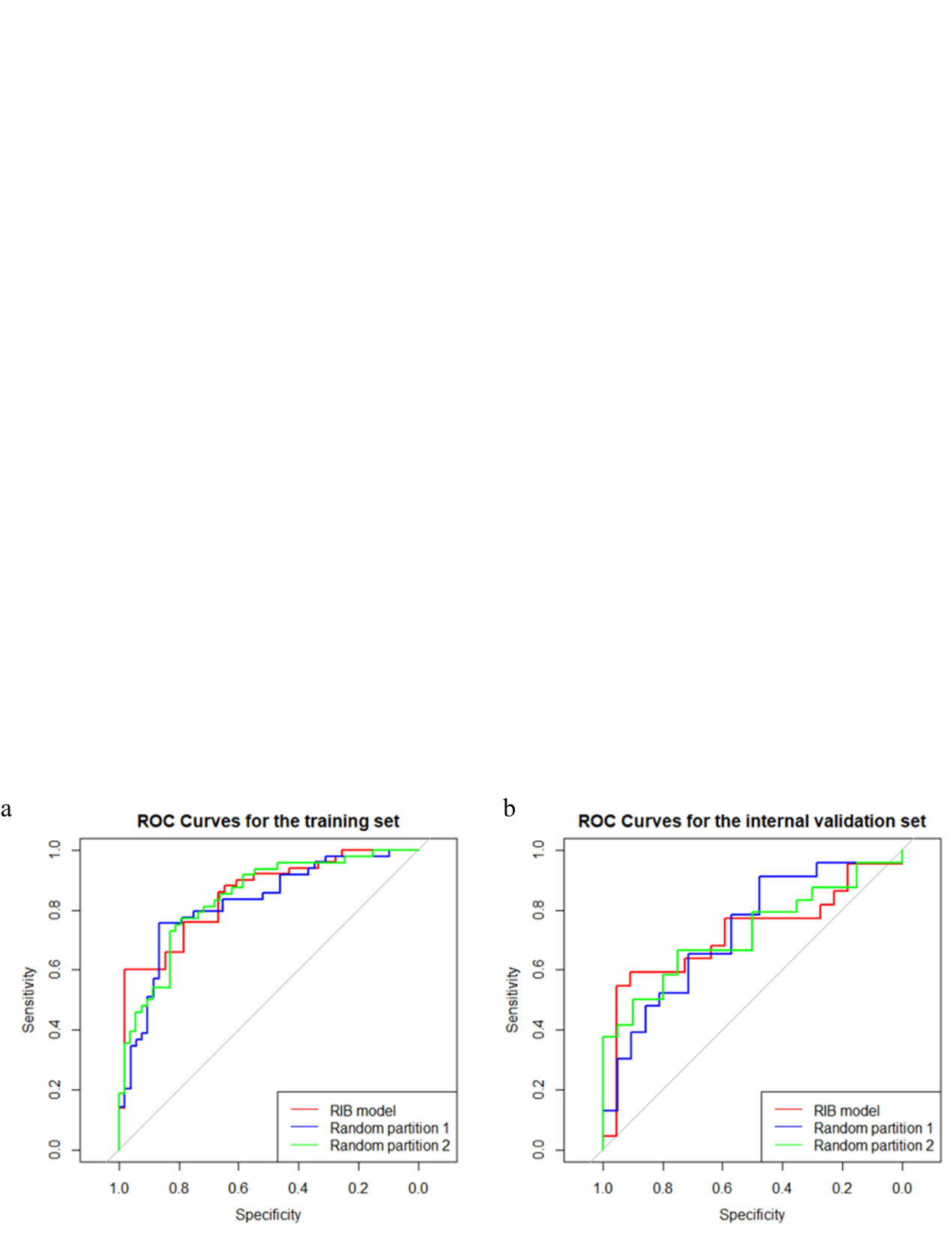


**Fig. S4** ROC curves for random partitions in the training set and internal validation set from TCGA microarray cohort showed the model is reliable.

(a) ROC curves in the training sets; (b) ROC curves in the internal validation sets.


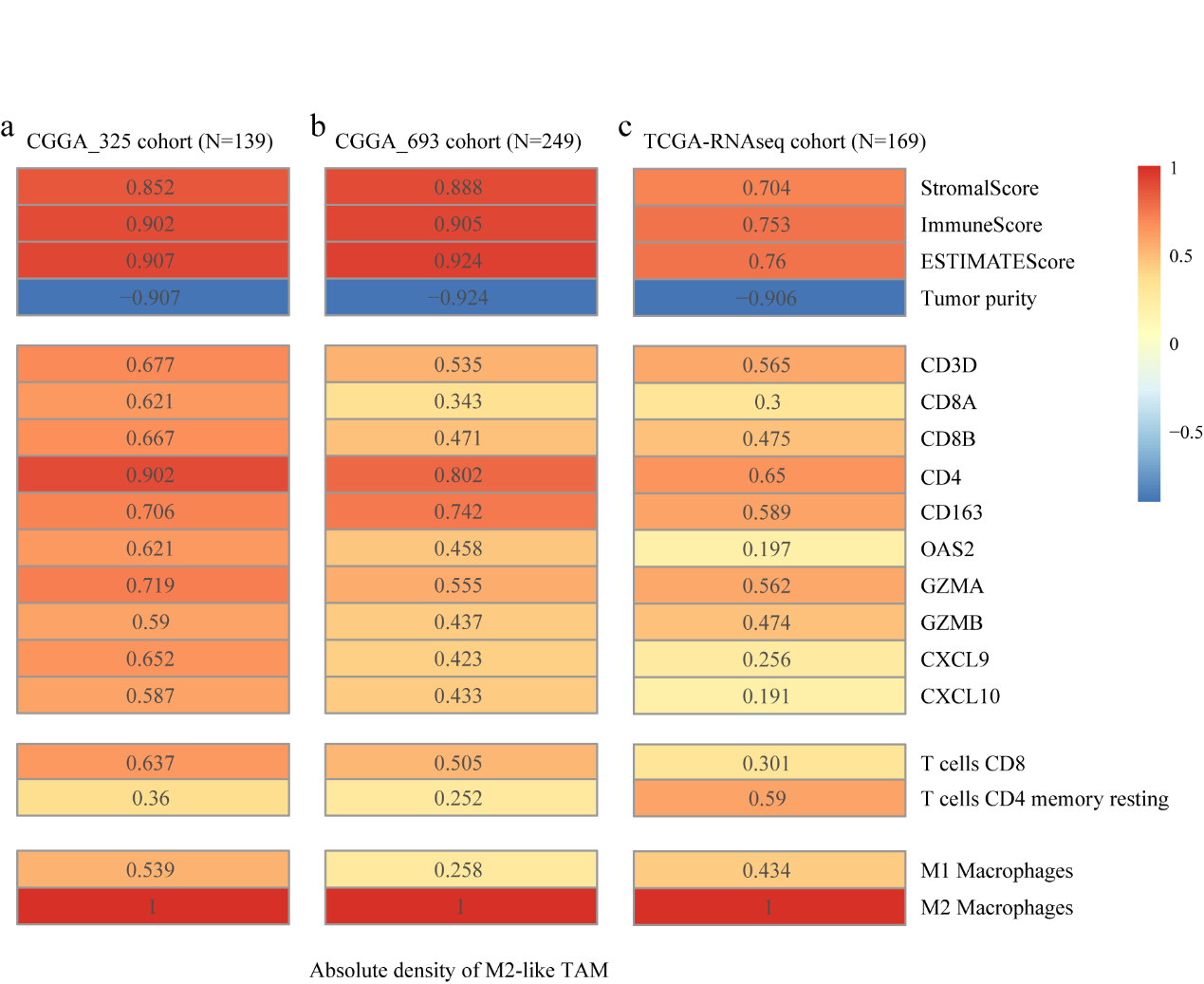


**Fig. S5** Estimated absolute fraction of M2-like TAM had positive correlation with immune signatures characterized in hot tumors (eg immunescore) as well as negative correlation with tumor purity characterized in cold tumors.

(a) CGGA_325 cohort; (b) CGGA_6935 cohort; (c) TCGA-RNAseq cohort.


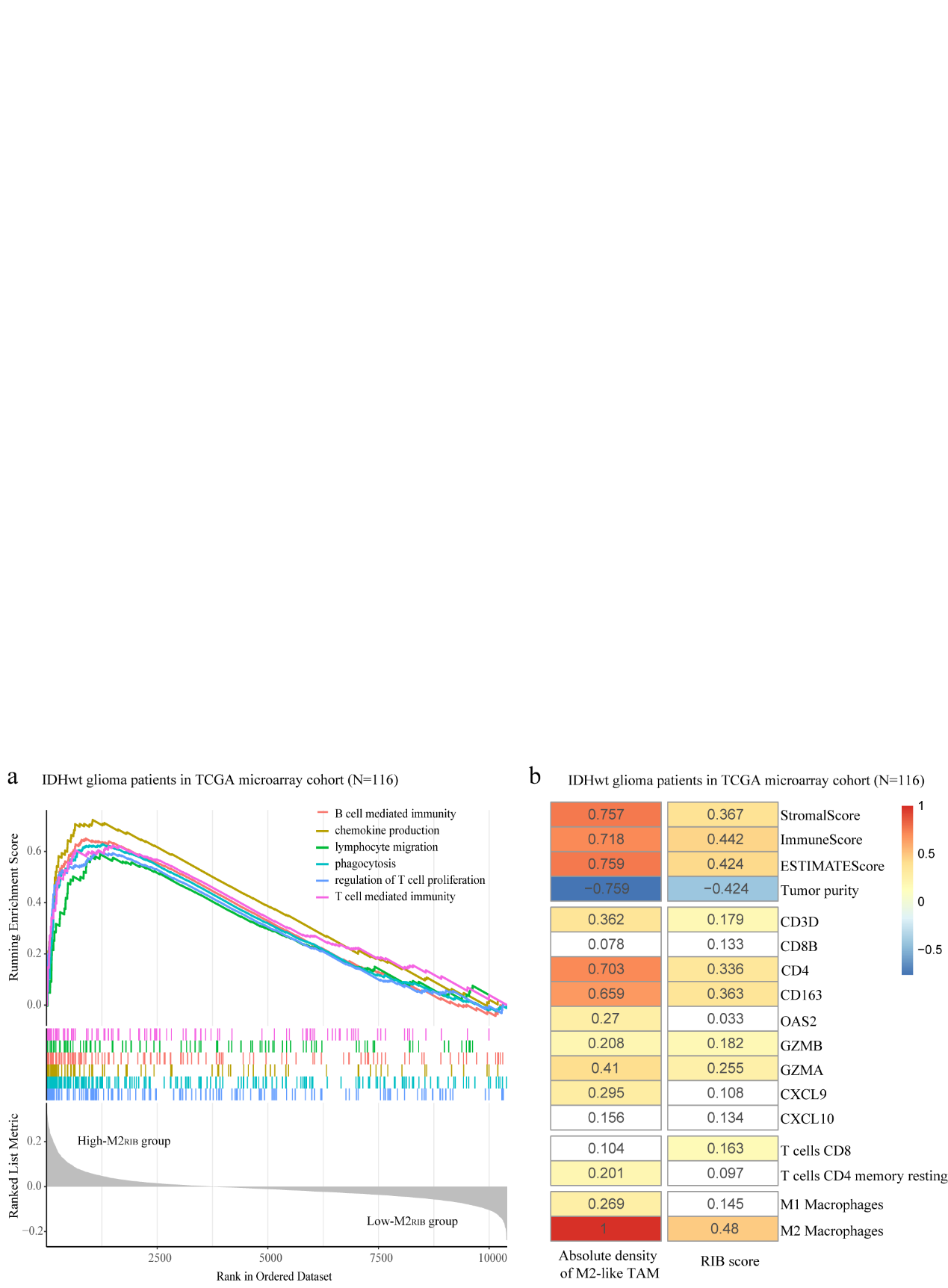


**Fig. S6** Biological analyses in IDH-wildtype glioma patients from TCGA microarray cohort showed positive relationship between RIB score and ''hot'' immunological microenvironment.

(a) GSEA enrichment analysis showed more extensive immune responses in patients from high-M2RIB group of IDH-wildtype glioma patients; (b) Both of the estimated absolute fraction of M2-like TAM (left) and RIB score (right) had positive correlation with immune signatures characterized in hot tumors (eg immunescore) as well as negative correlation with tumor purity characterized in cold tumors.


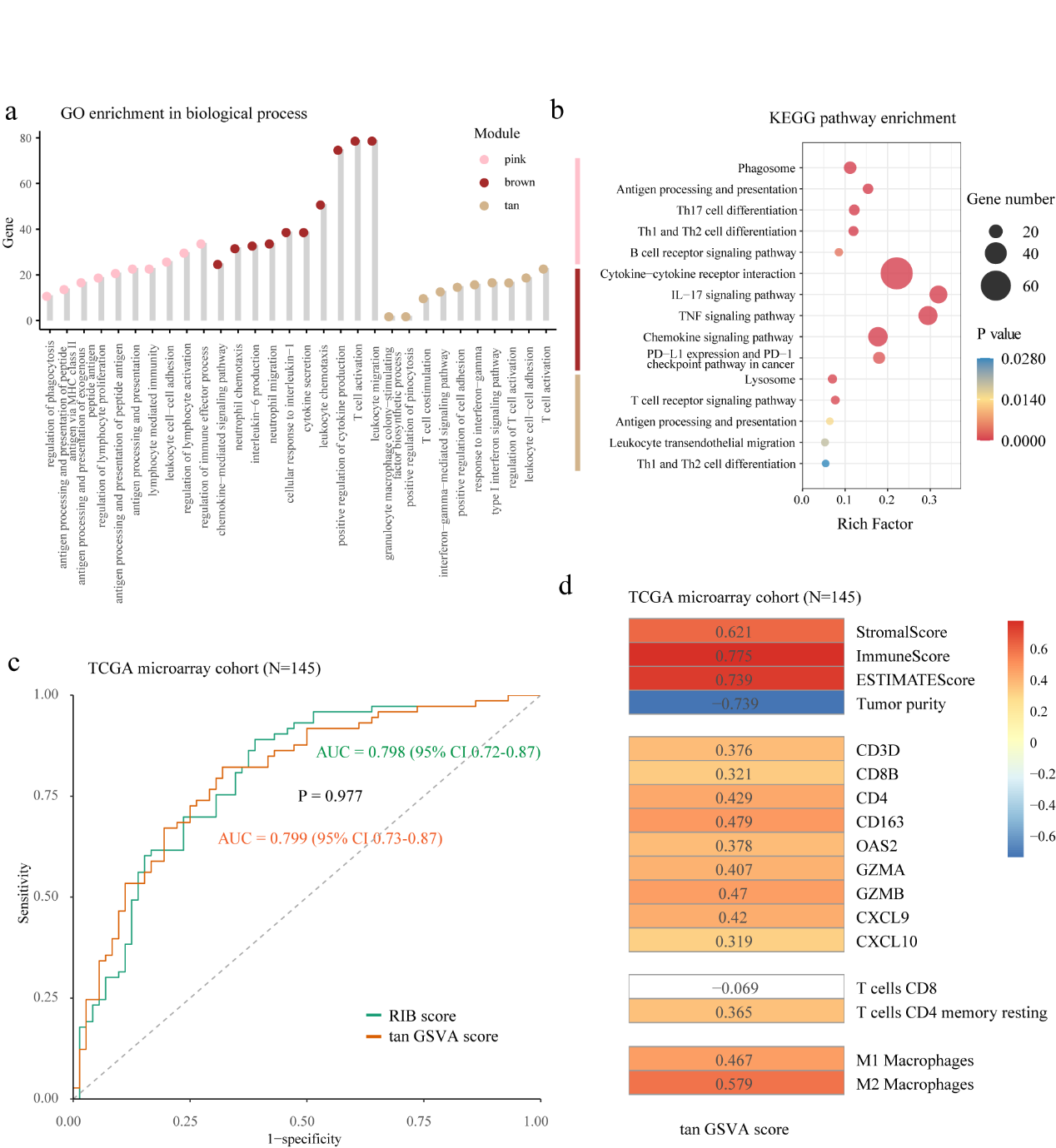


**Fig. S7** Gene module (tan) in close relationship with RIB score was enriched in immune response process especially triggered by macrophages.

(a) Gene Ontology enrichment analysis revealed that genes in three key gene modules were mainly distributed in process of immune responses; (b) The significant KEGG enrichment pathways related to immune response of genes in tan; (c) ROC curves also showed a high accuracy of GSVA enrichment score of tan gene module to predict density of M2-like TAM; (d) GSVA enrichment score of tan gene module had a highly positive correlation with immune signatures characterized in hot tumors.


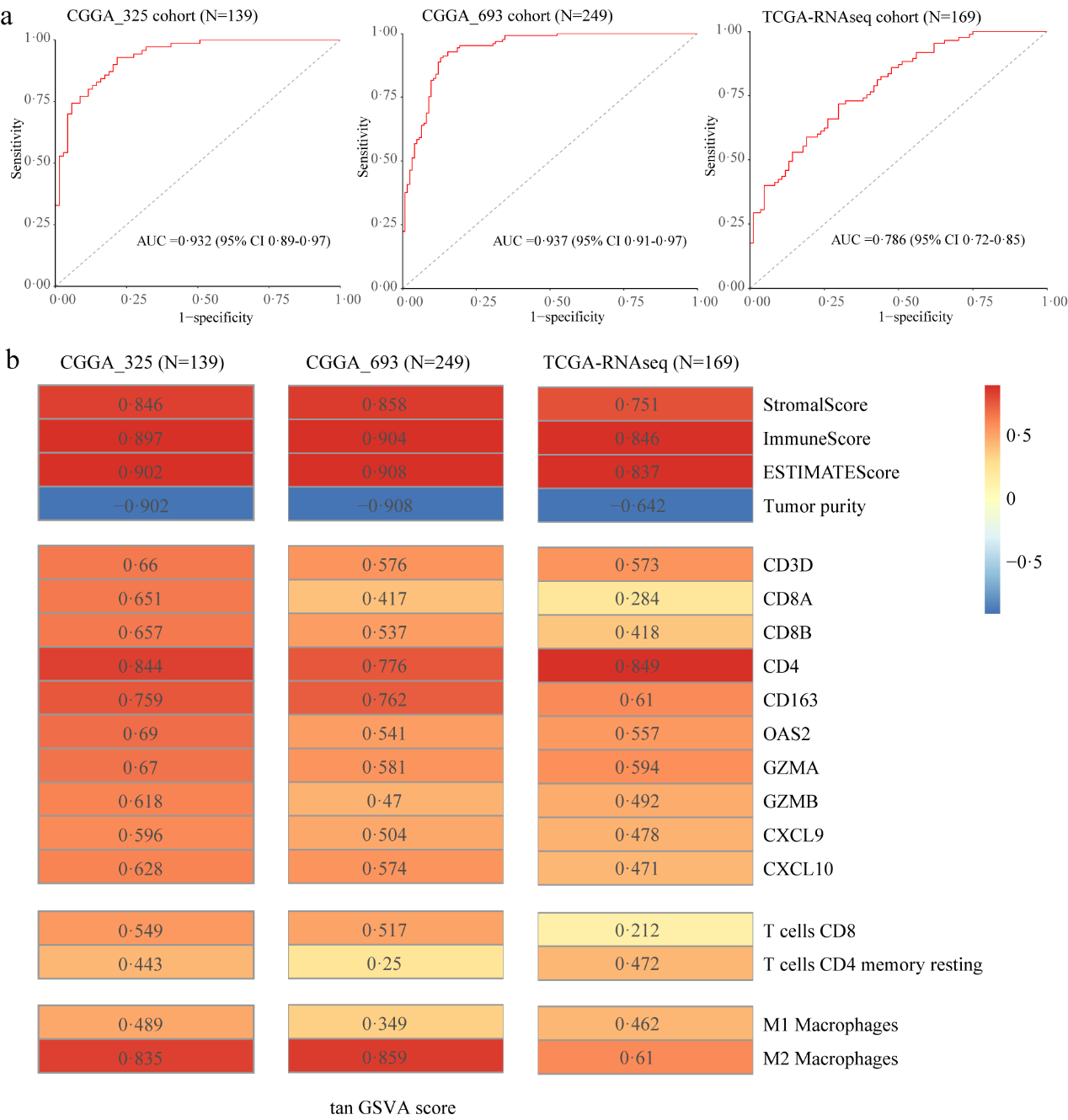


**Fig. S8** Gene module (tan) was in close relationship with hot immune-phenotype.

(a) ROC curves showed a high accuracy of GSVA enrichment score of tan gene module to predict density of M2-like TAM; (b) GSVA enrichment score of tan gene module had a highly positive correlation with immune signatures characterized in hot tumors.


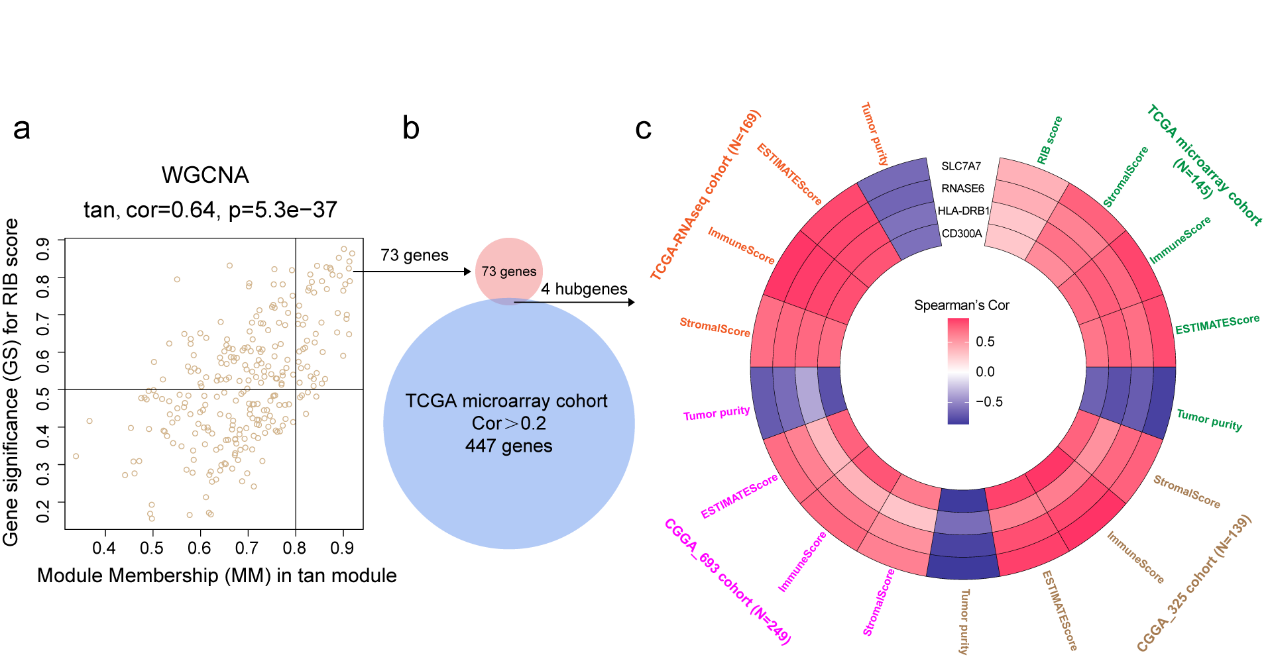


**Fig. S9** Gene module (tan) was in close relationship with hot immune-phenotype.

(a) Correlation between module membership and gene significance in the tan module; (b) Four significant genes highly correlated with RIB score were demonstrated; (c) All of the four hub-genes had highly positive correlation with immunescore characterized in hot tumors as well as significantly negative correlation with tumor purity characterized in cold tumors.


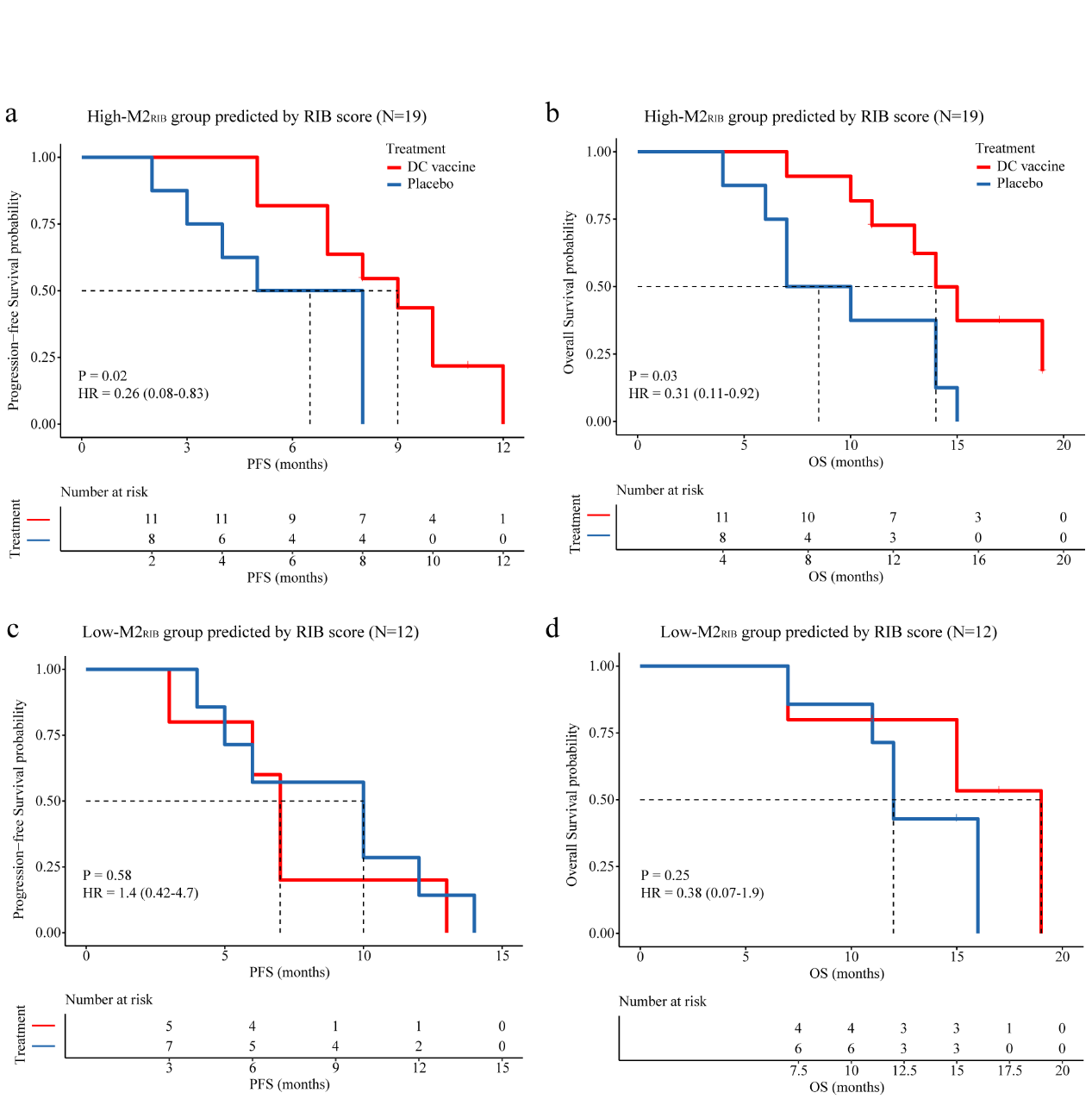


**Fig. S10** Kaplan-Meier analyses of progression-free survival (PFS) and overall survival (OS) according to the optimal cut-off value (0.65) in patients from immunotherapy-treated cohort.

(a) Patients with high RIB score (＞0.65) could prolong PFS by treatment from DC vaccine; b Patients with high RIB score (＞0.65) could prolong OS by treatment from DC vaccine; No significant survival differences of PFS (c) or OS (d) were found between patients treated by DC vaccine and placebo in subgroup with low RIB score (＜0.65).
