# Supplementary material for: MRI-derived radiomics assessing tumor-infiltrating macrophages enable prediction of immune-phenotype, immunotherapy response and survival in glioma
Source: Biomark Res. 2024 Jan 31;12:14. doi: 10.1186/s40364-024-00560-6 (PMC10829320; doi:10.1186/s40364-024-00560-6)
Supplement: Supplementary file 2 — Additional file 2. Supplementary Methods. [file 40364_2024_560_MOESM2_ESM.docx]

**Supplementary Methods**

**Immunohistochemistry (IHC) staining**

Immunohistochemistry for CD163 were performed as previously described[1]. Briefly, the formalin-fixed paraffin-embedded (FFPE) tumor tissue sections were deparaffinized followed by blocked with 3% H_2_O_2_ at room temperature for 20 min. Sections were retrieved by lycium hydrochloric acid (pH=6.0) and incubated overnight at 4℃ with CD163 antibody (Ab) (Abcam, ab182422) at dilution of 1:50. DAB Substrate kit (SK4100) was used to make color and Hematoxylin staining Solution was used to stain nuclei. The assessment of CD163 was assessed as the percentage of positive cells related to the total cell number as described in our previous study[1, 2].

**Multiplexed Immunostaining**

FFPE tumor tissue sections of two representative patients were stained with anti-CD8 Ab (Biolynx, BX50036, 1:100, China), anti-CD68 Ab (Biolynx, BX50031-C3, 1:400, China), anti-CD4 Ab (Abcam, Ab133616, 1:400, UK), anti-CD163 Ab (Abcam, Ab182422, 1:300, UK) and anti-CD3 Ab (Biolynx, BX50022-C3, 1:300, China) sequentially followed by manufacturer’s protocol. Slides were first deparaffinized and rehydrated, followed by microwave antigen retrieval using ER2 (Leica AR9961-CN, Germany). After blocking endogenous peroxidase, the primary Abs were applied sequentially. Leica Biosystems PowerVision Poly-HRP antimouse was used for secondary detection. Each site received an IHC Detection Kit containing following TSA fluorophores: TSA 620, TSA520, Neon 440, TSA 670, TSA 570, and spectral DAPI respectively. All fluorophores and DAPI were prepared according to manufacturer guidelines. After five sequential reactions, slides were counterstained with DAPI and observed at different scale.

**Construction of radiomic signature using LASSO logistic regression model**

In total, 841 radiomic features were extracted from T1CE sequence, and were subdivided into eight classes: 13 Shape feature, 18 First Order Features, 23 Gray Level Co-occurrence Matrix (GLCM) Features, 16 Gray Level Size Zone Matrix (GLSZM) Features, 16 Gray Level Run Length Matrix (GLRLM) Features, 5 Neighbouring Gray Tone Difference Matrix (NGTDM) Features, 14 Gray Level Dependence Matrix (GLDM) Features and 736 Wavelet Features.

The intraclass correlation coefficients (ICCs) of the interobservers and the intraobserver were computed to eliminate the radiomic features with low reproducibility. Intra-class correlation coefficients (ICCs) were used to assess the repeatability of radiomics feature extraction. We initially choose 25 random MRI images for whole-volume tumor segmentation and feature extraction. To assess interobserver repeatability, the whole-volume tumor segmentation was performed in a blinded way by two radiologists (JW with 9 years and JZ with 19 years experiences, respectively). Radiomic features with inter-observer ICCs greater than 0.75 were selected in our study. Feature selection was then conducted to eliminate high correlation features. When the variance of features was close to zero or their mean absolute correlation coefficient > 0.9, the features were eliminated to reduce redundancy[3]. Then, a feature selection method (minimum redundancy maximum relevance, mRMR) was used to identify the most discriminant feature subset from the remaining features and the highest-ranking features were selected from the feature subset[4]. After that, the Least Absolute Shrinkage and Selection Operator (LASSO) classifier was used to select the most predictive radiomic features from the top-ranking features and establish a radiomic model for predicting density of M2-like TAM[5]. The 4-fold cross-validation was used to determine the optimal parameter configuration of the LASSO on the training set.

**Tumor tissue acquisition, processing and RNA sequencing**

The fresh HGG tumor tissue from patients undergoing surgical resection were acquired after getting informed written consent. Tumor-derived macrophage/microglia were isolated as mentioned previously[6]. Briefly, samples were initially dissociated into fragments under sterile conditions using scissors. Then, tissue fragments were digested to be suspensions using papain. The suspensions were resuspended in cold sterile PBS and filtered through a 40-μm strainer cap, followed by centrifugation for 5 min at 300 g. CD11b^+^ cells were sorted using CD11b microbeads (Miltenyi Biotec, 130-093-634, Germany) followed by procedures provided by the manufacturer. Finally, the total RNA of CD11b^+^ cells were extracted and used for RNA sequencing at illumina platform. The fastq data of sequencing was processed as general pipeline, and the data in FPKM (Fragments Per Kilobase per Million) was generated for further bioinformatic analysis. The RNA-seq data are deposited in The National Omics Data Encyclopedia (NODE, https://www.biosino.org/node/, OEP003422).

**Bioinformatic analysis**

All of bioinformatic analysis were finished in R software. ESTIMATE (Estimation of STromal and Immune cells in MAlignant Tumour tissues using Expression data) algorithm[7] was used to calculate the tumor purity, stromal score and immune score. For absolute fraction of tumor-infiltrating immune cell, CIBERSORT (Cell-type Identification By Estimating Relative Subsets Of RNA Transcripts)[8] was used to predicted relative proportion firstly, which were corrected using tumor purity per sample[9]. Gene ontology (GO) term, KEGG pathway and gene set enrichment analysis (GSEA) were carried out in clusterProfiler packages[10].

In order to investigate the molecular microcosmic analysis of radiomic model and reveal the underlying association of radiomic model and immune phenotype, the weighted gene co-expression network analysis (WGCNA) was performed to determine genes that were correlated to RIB model and radiomic features[11]. First, all genes and samples in the expression profile passed the good genes or good samples test. The WGCNA package in R software was run to identify gene modules with highly similar expression data[12]. Then, the correlation matrix between the gene modules and the radiomic features of final model was built using Spearman rank correlations. The gene modules that correlated with radiomic features most significantly were selected as the key modules, which were used for subsequent function enrichment and signaling pathways analysis. For each gene modules, gene significance (GS) represents correlation level between expression pattern and radiomic features, and module membership (MM) represents correlation level between expression pattern and module eigengenes (MEs). In this study, genes with GS score >0.5 and MM score >0.8 were defined as hubgenes, which were used for subsequent analysis[13].

**References**

1. Chen D, Li G, Ji C, Lu Q, Qi Y, Tang C, Xiong J, Hu J, Yasar FBA, Zhang Y, et al: **Enhanced B7-H4 expression in gliomas with low PD-L1 expression identifies super-cold tumors.** *J Immunother Cancer* 2020, **8**.

2. Yao Y, Luo F, Tang C, Chen D, Qin Z, Hua W, Xu M, Zhong P, Yu S, Chen D, et al: **Molecular subgroups and B7-H4 expression levels predict responses to dendritic cell vaccines in glioblastoma: an exploratory randomized phase II clinical trial.** *Cancer Immunol Immunother* 2018, **67:**1777-1788.

3. Gillies RJ, Kinahan PE, Hricak H: **Radiomics: Images Are More than Pictures, They Are Data.** *Radiology* 2016, **278:**563-577.

4. Meng X, Xia W, Xie P, Zhang R, Li W, Wang M, Xiong F, Liu Y, Fan X, Xie Y, et al: **Preoperative radiomic signature based on multiparametric magnetic resonance imaging for noninvasive evaluation of biological characteristics in rectal cancer.** *Eur Radiol* 2019, **29:**3200-3209.

5. Huang YQ, Liang CH, He L, Tian J, Liang CS, Chen X, Ma ZL, Liu ZY: **Development and Validation of a Radiomics Nomogram for Preoperative Prediction of Lymph Node Metastasis in Colorectal Cancer.** *J Clin Oncol* 2016, **34:**2157-2164.

6. Müller S, Kohanbash G, Liu SJ, Alvarado B, Carrera D, Bhaduri A, Watchmaker PB, Yagnik G, Di Lullo E, Malatesta M, et al: **Single-cell profiling of human gliomas reveals macrophage ontogeny as a basis for regional differences in macrophage activation in the tumor microenvironment.** *Genome Biol* 2017, **18:**234.

7. Yoshihara K, Shahmoradgoli M, Martínez E, Vegesna R, Kim H, Torres-Garcia W, Treviño V, Shen H, Laird PW, Levine DA, et al: **Inferring tumour purity and stromal and immune cell admixture from expression data.** *Nat Commun* 2013, **4:**2612.

8. Newman AM, Liu CL, Green MR, Gentles AJ, Feng W, Xu Y, Hoang CD, Diehn M, Alizadeh AA: **Robust enumeration of cell subsets from tissue expression profiles.** *Nat Methods* 2015, **12:**453-457.

9. Wang Q, Hu B, Hu X, Kim H, Squatrito M, Scarpace L, deCarvalho AC, Lyu S, Li P, Li Y, et al: **Tumor Evolution of Glioma-Intrinsic Gene Expression Subtypes Associates with Immunological Changes in the Microenvironment.** *Cancer Cell* 2017, **32:**42-56.e46.

10. Yu G, Wang LG, Han Y, He QY: **clusterProfiler: an R package for comparing biological themes among gene clusters.** *Omics* 2012, **16:**284-287.

11. Zhang B, Horvath S: **A general framework for weighted gene co-expression network analysis.** *Stat Appl Genet Mol Biol* 2005, **4:**Article17.

12. Langfelder P, Horvath S: **WGCNA: an R package for weighted correlation network analysis.** *BMC Bioinformatics* 2008, **9:**559.

13. Zhou Y, Fu X, Zheng Z, Ren Y, Zheng Z, Zhang B, Yuan M, Duan J, Li M, Hong T, et al: **Identification of gene co-expression modules and hub genes associated with the invasiveness of pituitary adenoma.** *Endocrine* 2020, **68:**377-389.
